# Reporting of harms outcomes: a comparison of journal publications with unpublished clinical study reports of orlistat trials

**DOI:** 10.1186/s13063-016-1327-z

**Published:** 2016-04-22

**Authors:** Alex Hodkinson, Carrol Gamble, Catrin Tudur Smith

**Affiliations:** Centre for Reviews and Dissemination, University of York, Heslington, York, YO10 5DD UK; Department of Biostatistics, MRC North West Hub for Trials Methodology Research, University of Liverpool, Liverpool, England, UK

**Keywords:** Harms, Orlistat, Clinical study report, Adverse event, Adverse effect, Randomised controlled trial, Systematic review, Evidence-based healthcare, Obesity

## Abstract

**Background:**

The quality of harms reporting in journal publications is often poor, which can impede the risk-benefit interpretation of a clinical trial. Clinical study reports can provide more reliable, complete, and informative data on harms compared to the corresponding journal publication. This case study compares the quality and quantity of harms data reported in journal publications and clinical study reports of orlistat trials.

**Methods:**

Publications related to clinical trials of orlistat were identified through comprehensive literature searches. A request was made to Roche (Genentech; South San Francisco, CA, USA) for clinical study reports related to the orlistat trials identified in our search. We compared adverse events, serious adverse events, and the reporting of 15 harms criteria in both document types and compared meta-analytic results using data from the clinical study reports against the journal publications.

**Results:**

Five journal publications with matching clinical study reports were available for five independent clinical trials. Journal publications did not always report the complete list of identified adverse events and serious adverse events. We found some differences in the magnitude of the pooled risk difference between both document types with a statistically significant risk difference for three adverse events and two serious adverse events using data reported in the clinical study reports; these events were of mild intensity and unrelated to the orlistat. The CONSORT harms reporting criteria were often satisfied in the methods section of the clinical study reports (70–90 % of the methods section criteria satisfied in the clinical study reports compared to 10–50 % in the journal publications), but both document types satisfied 80–100 % of the results section criteria, albeit with greater detail being provided in the clinical study reports.

**Conclusions:**

In this case study, journal publications provided insufficient information on harms outcomes of clinical trials and did not specify that a subset of harms data were being presented. Clinical study reports often present data on harms, including serious adverse events, which are not reported or mentioned in the journal publications. Therefore, clinical study reports could support a more complete, accurate, and reliable investigation, and researchers undertaking evidence synthesis of harm outcomes should not rely only on incomplete published data that are presented in the journal publications.

**Electronic supplementary material:**

The online version of this article (doi:10.1186/s13063-016-1327-z) contains supplementary material, which is available to authorized users.

## Background

There are two driving concerns that continue to grow when relying on published medical research to reflect the truth [1]. First, trials often remain unpublished years after completion, and the results are, therefore, unavailable to the public. Second, trials often display a distorted representation, where publications present a biased or misleading description of the design, conduct, or results of a trial [2, 3].

Journal publications and registry reports currently represent the main information source for obtaining summaries of clinical trial data for the purposes of clinical and health policy decision-making [4]. Results in the past have found reporting in journal publications to be inadequate and inconsistent [5], and although clinical trial registries have been responsible for making major strides in improving the transparency of trial data, a recent study suggested that the results from trial registries often remain unavailable [6].

The clinical study report (CSR) is a structured document that summarises the analysis methods and results of a clinical trial submitted for marketing authorization of an investigational medicinal product in the European Union, Japan, or the United States. CSRs are an ‘integrated’ full report, which can be up to a thousand pages in length, and include extensive detailed information on the efficacy and harms of interventions. The information in these documents relating to harms is usually separated individually by adverse event (AE) and serious adverse event (SAE) terms in summary tables and listings.

In the past, researchers have made major efforts to gain access to CSRs, with the intention to inform regulatory decision-making [7]. The information contained in the CSRs has proved vital when evaluating both the efficacy [8] and safety [9] of clinical interventions. Evidence from journal publications has previously been questioned, and even overturned, by findings from unpublished information reported in the CSR [10]. In December 2009, Roche was the first global healthcare company to release ‘Clinical Study Reports’ after growing concerns over their product Tamiflu [8]. Their policy now allows researchers to access the CSRs and summary reports used for regulatory purposes since 1 January 1999. In 2010, the European Medicine Agency (EMA) [11] became the first major regulatory agency to agree to an open-access policy to confidential documents, including CSRs. However, in 2013, the EMA was forced to step backwards when the general court of the European Union (EU) ordered them to limit the access to their reports due to legal cases from two drug companies [12]. In October 2014, the EMA published their final policy on the access to documents and CSRs [13].

Orlistat (trade name: Xenical) is marketed by Roche in most countries. It is used in the treatment of obesity as a selective inhibitor of gastric and pancreatic lipase [14]. Mild, but unpleasant, gastrointestinal (GI) side effects are commonly reported with orlistat use. A recent review [15], including 16 randomized placebo-controlled trials of orlistat, estimated an increased risk of discontinuations due to AEs of 3 % (95 % CI 1–4 %) with orlistat. The most common AEs leading to withdrawal were GI (40 %); only eight (50 %) trials specified the number of AEs due to GI problems. Another study [16] of 29 trials of orlistat indicated an increase in the risk for diarrhoea, flatulence, abdominal pain, and dyspepsia in orlistat-treated patients compared with placebo. No SAEs were reported in these reviews. Concern exists that there may also be an associated increased risk of serious hepatic events, as indicated in a case series study using primary care data from the Clinical Practice Research Datalink (CPRD) [17].

We aim to carry out an exploratory review consisting of two main analyses: (1) to compare the number(s) of reported harms (AEs and SAEs) and (2) the structured reporting of harms. Both analyses will be assessed between CSRs and journal publications using a case study of Roche-sponsored orlistat trials to provide a summary of the added value, if any, from the CSRs. To our knowledge, an in-depth exploration that includes a detailed meta-analysis of this type has not been published in previous CSR-related research.

## Methods

We planned to identify independent trials, each of which were reported within two different trial summary reports: CSRs and publically available journal publications. The aim was to compare these document types and determine whether there were inconsistencies in the quality and quantity of reporting of harms. The CSRs were released by Roche (Genentech; South San Francisco, CA, USA).

### Identifying the studies

A search was implemented by one researcher (AH) in the Cochrane Central register (final search 6 July 2013) and Ovid MEDLINE (final search 2 July 2013) to obtain all relevant published, randomised, controlled trials comparing orlistat against a placebo for obesity treatment. The search strategies are provided in Additional file 1. Each full article was assessed independently by one reviewer (AH) to determine eligibility. We included published and unpublished RCTs investigating the use of orlistat. No restriction was placed on the clinical area. Observational studies and those studies that did not specify orlistat as their primary intervention were excluded.

### Data collection and extraction

Roche was contacted and asked to provide the corresponding CSRs for each of the publications identified. A Roche CSR consists of the following five modules of information:

- Module I: The ‘Core report’ – background and rationale, objectives, materials and methods, efficacy results, safety results, discussion, conclusion and appendices
- Module II: ‘Study documents’ – protocol and amendment history, blank case report forms (CRFs), subject information sheet and consent form, glossaries of original and preferred terms, randomization list, reporting analysis plan (RAP), certificates of analysis, list of investigators and list of ethics committee members
- Module III: ‘Listings of demographic and efficacy data’
- Module IV: ‘Listing of safety data’
- Module V: ‘Statistical report and appendices’ – statistical analysis and efficacy results

For each matching document pair (CSR and journal publication), the following data were extracted:

- Content and characteristics of both document types, including whether a clear primary objective of safety was defined, a word count of the information relating to harms in both the journal publication (including any online supplementary material) and in the CSR documents of text only (word count performed using the software AnyCount version 7.0 [18]). Missing pages relating to safety due to redactions were noted in the results; we managed to obtain these on further request.
- Name of each reported AE and SAE term recorded for both placebo and orlistat, with the overall number of patients in the safety population, as defined in the respective document. The intensity grading (i.e. mild, moderate, or severe), relationship to orlistat, and definition of the SAEs were also observed where possible. SAEs were defined as any event that was fatal or life-threatening, requiring hospitalization or prolongation of hospitalization, or an overdose. The AE coding system was also detailed.
- Reporting structure of harms (CONSORT-harms [19] used as a benchmark). The CONSORT extension for reporting harm outcomes extends ten checklist items of the CONSORT (2001) checklist to help support the reporting of harms-related data from RCTs. This includes guidance on how to report harms in the title and abstract, introduction, methods (definitions, collection, and analysis), results (withdrawals, denominators, and type), and the discussion.

One researcher (AH) extracted, and a second reviewer (CTS) checked the data extraction. Discrepancies in the rates of agreement were resolved through consensus or recourse to a third reviewer (CG), where necessary. As there were no disagreements in the data extraction for the first three trials (NM16189, M37013, and M37002), extraction for the final two trials was only carried out by one reviewer (AH).

### AEs and SAEs

For a particular trial, all harms (AEs and SAEs) reported in either the journal publication or the CSR were extracted and compared across the two document types. The clinically validated medical terminology dictionary MedDRA is commonly used during the regulatory process by all stakeholders in healthcare; it is used for coding harm outcomes. These reported outcomes were then organized into each of the five levels of the MedDRA dictionary: the system organ class, high-level group term, high-level term, preferred term, and lowest level term. Outcomes are usually reported in the journal publications and CSRs as MedDRA preferred term level events. Therefore, we compared the total number of reported MedDRA preferred terms, and if a preferred term was reported in both the CSR and journal publication, the numerical data were compared, and any discrepancies, noted.

For each MedDRA preferred term (AE and SAE), the data extracted from the CSRs were used to estimate risk differences, which were pooled across trials using fixed-effect meta-analysis. A corresponding meta-analysis was performed using the data extracted from the journal publications wherever relevant. The pooled risk difference (RD) with 95 % confidence interval [20] and the I^2^ statistic [21] were compared between the CSR-based and the journal publication-based analyses. As the SAE data were sparse, a sensitivity analysis was undertaken to pool the relative risk (RR). We stress that these meta-analysis results are based on a subset of the eligible trials of orlistat and are presented for the purpose of methodological comparison rather than definitive clinical results.

### Structured reporting of harms

Using the CONSORT-harms extension [19] as a benchmark for reporting harms data from a randomised controlled trial, documents were assessed across 15 adapted criteria (see Table 1) that focus on the methods and results. Each trial was classified as follows for each individual criteria:

**Table 1:** Fifteen criteria (adapted from the CONSORT-harms extension) assessed to evaluate the completeness of reporting methods and results of harms

	Criteria	Criteria description	Description of complete reporting for criteria
Methods	1	List addressed adverse events with definitions	Listed AEs with definitions (with attention to the grading, when relevant)
	2	Mode for collecting data	Full description of questionnaires, interviews, or tests used to collect information on the harms. Detailed information on the questions asked
	3	Timing and time frame of surveillance	Description of the time frame of surveillance for AEs, with the stopping period detailed
	4	Attribution methods	Person responsible for making attribution disclosed and whether blinding was used
	5	Intensity of ascertainment	Specify clearly how the withdrawals are handled in the analyses
	6	Harms-related monitoring	Plans for monitoring and rules for stopping for the benefits and harms separately
	7	Coding of AEs	Reference to any coding system used and person responsible for the coding
	8	Handling of recurrent events	Specify how recurrent events are handled: detailed as separate events or as one
	9	Timing issues	Timing of events explained, if recurrent
	10	Plans to perform any statistical analyses and inferences	Described how pre-specified statistical analyses are separated from post hoc analyses, and any common problems addresses
Results	11	Withdrawals and discontinuations	Reasons for discontinuations and separated by arm. Flow diagrams used to display withdrawals
	12	Denominators for analyses on harms	Analyses and definitions used and clearly stated (i.e. intention to treat (ITT)), and all denominators for safety population are clearly detailed
	13	Specifying AE type	Results presented separately by System Organ Classification type
	14	Grading or scaling used	Each AE type should offer appropriate metrics of absolute risk
	15	Seriousness per arm	Reported separately for each type of event

- BOTH – both documents report the criteria
- CSR – only reported criteria in the clinical study report
- Pub – only reported criteria in the trial publication
- NR – criteria not reported in either document

The total number of criteria satisfied in each CSR and journal publication for a particular trial was calculated and expressed as a percentage of 15 criteria.

When both document types reported on any particular individual criteria (i.e. BOTH), the reported information was compared and classified as follows:

- CSR (+) – The CSR provides more information than the journal publication
- Similar (O) – Both document types provide equal and similar information
- CSR (-) – The journal publication provides more information than the CSR

## Results

**Fig. 1:**
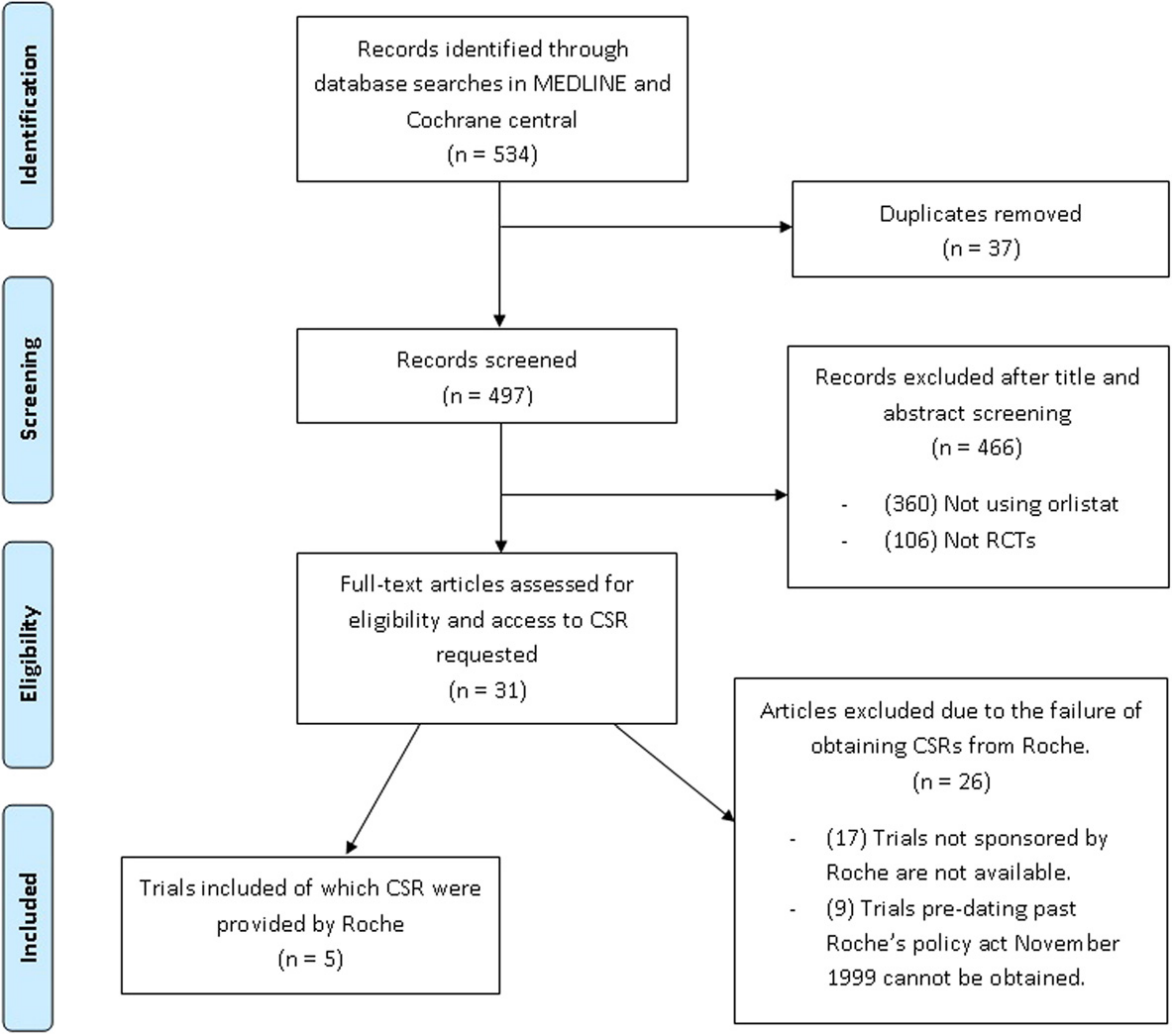
Flow diagram for obtaining the trial reports

Thirty-one journal publications related to 31 randomised controlled trials of orlistat were identified in our search (Fig. 1). We requested access to the full CSRs from Roche corresponding to each of these trials. The CSRs could not be provided for 26 of these trials. Of the 26 trials, 17 were not Roche-sponsored, and therefore, the CSRs were not held by Roche. Nine trials pre-dated Roche’s policy extension, which only allows access to trials dating back to 1 January 1999.

CSRs were obtained and matched with the corresponding journal publication for five trials (NM16189 [17], M37013 [18], M37002 [19], M37047 [20], and BM15421 [21]). Module I of the CSR was provided for all trials. Module II was not provided for one trial (BM15421), and module V was not provided for one trial (NM16189). We contacted Roche to provide reasons for these missing modules and for the four missing pages. Roche informed us that these sections contained confidential information and had to be removed. Modules III and IV were not provided for any of the trial CSRs because they contained individual patient data listings.

Table 2 shows the content and characteristics for each trial document pair. Safety was not the primary objective for any of the five trial journal publications but was defined as a secondary objective in three journal publications [22–24] and was not specified in two journal publications [25, 26]. Two trials [23, 25] were published in the Journal of Diabetes, Obesity and Metabolism; two trials [24, 26], in the Journal of Diabetes Care; and one trial [22], in the Journal of the American Medical Association (JAMA).

**Table 2:** Content and characteristics of the trial documents

Trial ID	NM16189		M37013		M37002		M37047		BM15421	
Safety primary objective of trial?	No†		No†		No¥		No¥		No†	
Journal publication: author, journal and year	Chanoine [22], Journal of the American Medical Association (JAMA (2005)		Halpern [23], Diabetes, Obesity and Metabolism (2003)		Hanefeld [25], Diabetes, Obesity and Metabolism (2002)		Kelley [26], Diabetes Care (2002)		Torgerson [24], Diabetes Care (2004)	
CSR research report no. (date of CSR)	1011426 (2003)		1002688 (2000)		1003882 (2001)		1002743 (2001)		1008213 (2002)	
Word count (including text and numbers, but not tables)										
Trial document	Pub	CSR	Pub	CSR	Pub	CSR	Pub	CSR	Pub	CSR
Total number of words in document^ϵ^	10,568	146,801	6,371	45,464	6,382	140,166	7,090	170,347	5915	314,277
Total number of words relating to safety (% of total)	1,147 (10.9)	4,883 (3.3)	908 (14.3)	2,664 (5.9)	638 (10)	4,964 (3.5)	707 (10)	4,150 (2.4)	387 (6.5)	6,653 (2.1)
CSR Module^ɸ^ supplied by Roche										
I	✓^Π^		✓^Π^		✓		✓^Π^		✓^Π^	
II	✓		✓		✓		✓		*	
III	*		*		*		*		*	
IV	*		*		*		*		*	
V	*		✓		✓		✓		✓	

Footnote:

*CSR* Clinical study report, *Pub* Journal publication

†Safety secondary objective in both the CSR and journal publication; ¥Objective to assess improvements in glycaemic control and cardiovascular disease risk in both CSR and Journal publication; ^**ɸ**^ **Module: I** = Core report (background and rationale, objectives, materials and methods, efficacy results, safety results, discussion, conclusion and appendices); **II** = Study documents (protocol and amendments history, black case report form (CRF), subject information sheet and consent form, glossaries of original and preferred terms, randomization list, reporting analysis plan (RAP), certificates of analysis, list of investigators, list of ethics committee members); **III** = Listing of demographic and efficacy data; **IV** = Listing of safety data; **V** = Statistical reports and appendices (Statistical analysis, efficacy results). ✓Module provided in CSR; *Roche did not provide these modules, since they contained individual patient data listings and therefore were deleted. ϵ We could only count words for modules that were made available by Roche, so the actual number would be greater than this. The percentage of words relating to harms would therefore differ; ^Π^ CSRs each had one missing page in module I, which Roche provided upon further requests. Any additional information from this was used in the results.

The mean word count across the five trial journal publications was 7,265 (standard deviation (sd) 1,894), with an average of 10 % of words (mean (sd) 757 (287)) dedicated to safety. The CSRs had a mean (sd) of 163,411 (96,872) words across all trials, with approximately 3 % (mean (sd) 4,663 (1,446)) related to safety. The mean difference between the CSR and journal publication was 3,906 (95 % CI (1,756; 6,056)) words.

### Comparison of reported AE and SAE event data

MedDRA version 2.3 had been used to code AEs and SAEs in all five trials.

### Adverse events

**Fig. 2:**
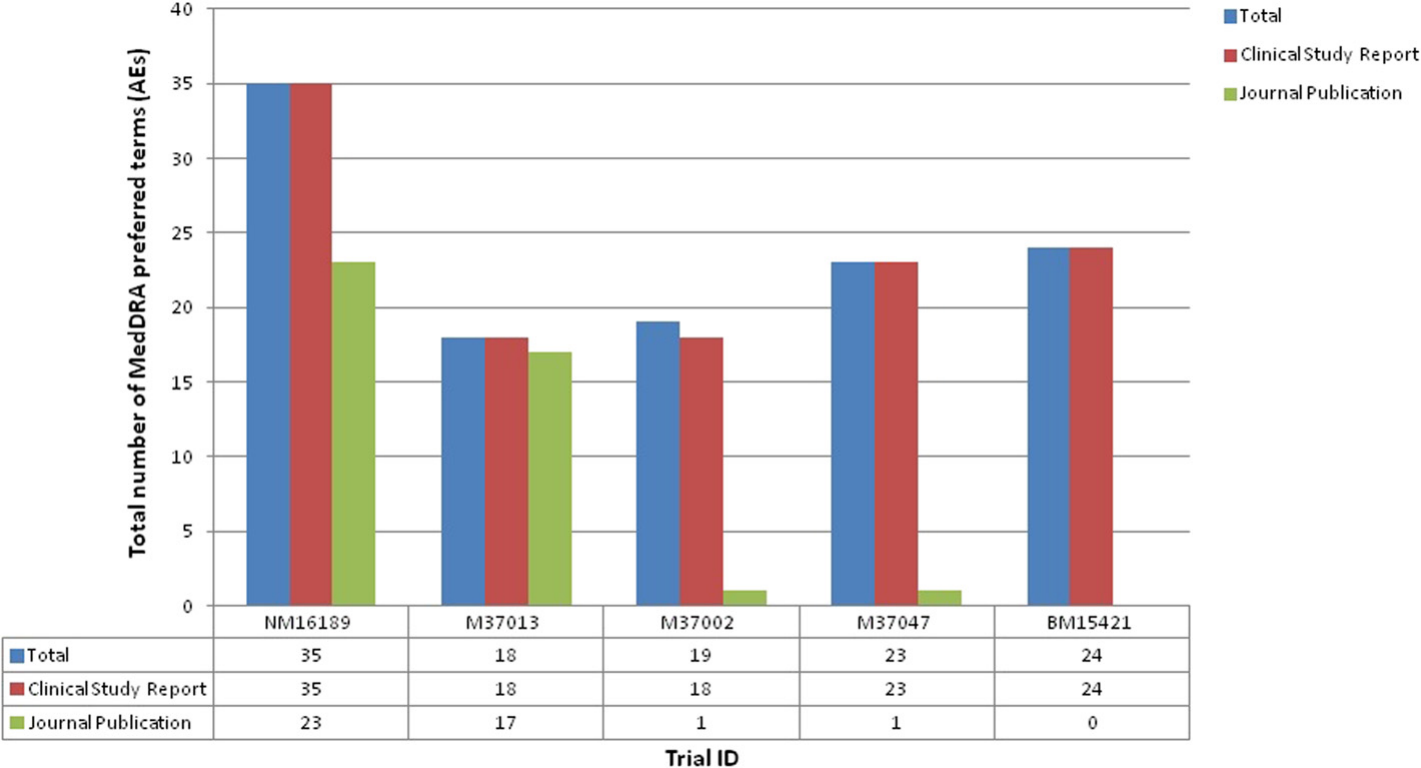
The total number of MedDRA preferred terms (Adverse Events) reported in clinical study reports (CSRs) and journal publications across all five trials. Footnote: Total: Total number of individual MedDRA preferred terms related to AEs reported across the CSR and journal publication for a trial

The total number of MedDRA preferred terms for AEs varied across trials (Fig. 2) (Forest plots are provided in Additional file 1). The journal publications did not always report the complete list of terms identified in the corresponding CSR, but all of these ‘missing’ AEs were of mild to moderate intensity and were unrelated to the intervention. For instance, in one trial (M37013), very good consistency in reporting was observed between the CSR and journal publication, with 18 AEs reported in total, 18 (100 %) of which were listed in the CSR and 17 (94 %) in the journal publication. However, very poor consistency was observed for the three trials (M37002, M37047, and BM15421), with 5 % or fewer of the total AEs being reported in the journal publication (M37002, one (5 %); M37047, one (4 %); BM15421, 0 (0 %)). When a MedDRA preferred term was listed in both the CSR and journal publication, complete agreement was observed in the numerical results (Additional file 2) except for one trial (M37013), where three additional patients with ‘abdominal pain’ on orlistat were identified within the journal publication.

In the meta-analysis (MA) for the AEs (Table 3), 61 individual MedDRA preferred terms were reported in either the CSR or journal publication across the five trials (Additional file 1). Thirty (49 %) of these terms were reported in the CSR and corresponding journal publication for at least one trial, thereby allowing a comparison of the pooled results. In six (20 %) of the 30 MA comparisons, the magnitude of the effect differed (the 95 % CI for the pooled risk difference (RD) did not overlap between the CSR and the journal publication results). These include the AE terms: ‘increased defecation’, ‘oily spotting’, ‘oily evacuation’, ‘faecal incontinence’, ‘soft stools’, and ‘faecal urgency’. For the 31 AE terms that had only been reported in a CSR, 23 (74 %) analyses suggested an increased risk of an AE on orlistat, two (6 %) of which were statistically significant (faeces discolouration and dry skin); these AEs were mild and were unrelated to treatment. For four (13 %) terms, an increased risk of an event occurred with the placebo, one (3 %) of which was statistically significant (haemorrhoids) and of a mild grade.

**Table 3:** Summary of meta-analysis results for the individual MedDRA preferred term adverse events pooled across all five trials

Adverse events (AEs)		Breakdown of adverse events reporting		
Meta-analysis characteristic	Total	Once in the clinical study report (CSR) and journal publication	CSR	Journal publication
Number of AE terms reported (% of total)	61	30 (49 %)	31 (51 %)	0 (0)
Direction of pooled risk effect in meta-analysis		For all 30 AEs there is agreement in direction of the pooled risk effect between the pairing of documents	• 23 (74 %) showed an increased pooled risk of AE on orlistat • four (13 %) showed no difference • four (13 %) showed increased pooled risk of AE on placebo	
AE listings for when there is a change in effect including statistical significance		• Pooled risk effect was greater in journal publication for four AEs; *increased defecation, oily spotting, oily evacuation, faecal incontinence* • Pooled risk effect was greater in the CSR for two AEs; *soft stools, faecal urgency*	• two (6 %) of the 23 AEs were statistically significant; *faeces discolouration, dry skin* ^a^ • one (3 %) of the four AEs with increased risk on placebo was statistically significant; *haemorrhoids* ^a^	

Footnote:

^a^These adverse events were mild and unrelated to treatment

### Serious adverse events

**Fig. 3:**
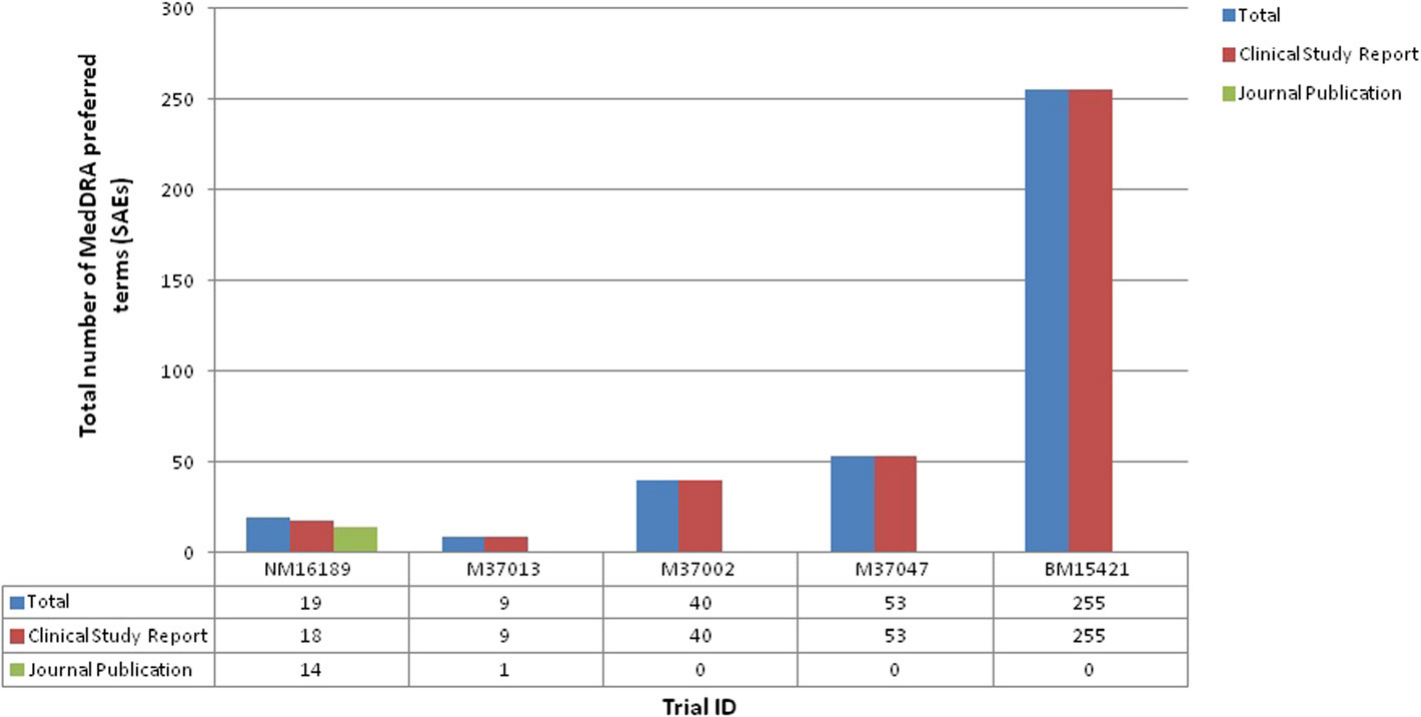
The total number of serious adverse events reported in the clinical study reports (CSRs) and journal publications across all five trials. Footnote: Total: Total number of individual MedDRA preferred terms related to SAEs reported across the CSR and journal publication for a trial

The total number of MedDRA preferred terms for SAEs were generally poorly reported in journal publications (Fig. 3; Additional file 3). For the four trials (M37013, M37002, M37047, and BM15421) only 11 % or fewer of the total SAE terms were reported in the journal publication with 11 %, 0 %, 0 %, and 0 %, respectively. All SAEs that were reported only in the CSR were of mild intensity grading and were unrelated to the treatment.

In trial NM16189, 19 SAE terms were reported across the CSR and journal publication. Thirteen of these were reported in both documents, either with full numerical agreement (12 SAE terms) or with disagreement in numerical results (one depression SAE on orlistat reported in the CSR, and two depression SAEs reported in the journal publication) (Additional file 3). Five SAE terms were only reported in the CSR (demyelination (one) and bronchospasm aggravated (one) on placebo, and convulsions (one), suicidal ideation (one) and liquid stools (one) on orlistat). Encephalomyelitis as an SAE was reported for placebo in the publication but not the CSR. Trial M37013 reports nine SAEs, with only “diarrhoea and dehydration” on orlistat reported in both documents. The remaining eight SAEs were only reported in the CSR; death (one), diabetes mellitus (one), hysterectomy and perineoplasty (one), mitral lesion (one) on placebo and Chronic cholecystitis (one), nephrectomy due to previous renal carcinoma (one), nephrectomy and lithotripsy due to previous nephrolithiasis (one), ovary carcinoma and ascites (one) on orlistat. The three remaining trials (M37002, M37047, and BM15421) report a high number of SAEs (40, 53, and 255) within the CSR that have not been reported in the corresponding journal publication.

In the MA for the SAEs (Table 4), 326 individual terms were reported in either the CSR or journal publication across the five trials (Additional file 4). Fourteen (4 %) of these terms were reported in the CSR and corresponding journal publication for at least one trial, allowing a comparison of the pooled results. For the 311 (95 %) terms that had only been reported in a CSR, 16 (5 %) analyses suggested an increased risk of an SAE on orlistat, two (13 %) of which were statistically significant (carotid artery stenosis and varicose veins), but all were mild and unrelated. In the sensitivity analysis, pooling relative risk rather than risk differences, no SAEs were found to be statistically significant. However, we were unable to estimate the pooled relative risk for ten AEs (including carotid artery stenosis and varicose veins), as they include multiple studies reporting no events in the placebo group.

**Table 4:** Summary of meta-analysis results for the individual MedDRA preferred term serious adverse events pooled across all five trials

Serious Adverse Events (SAEs)		Breakdown of serious adverse events reporting		
Meta-analysis characteristic	Total	Once in the clinical study report (CSR) and journal publication	CSR	Journal publication
Number of SAE terms reported (% of total)	326	14 (4 %)	311 (95 %)	1 (<1 %)
Direction of the pooled risk effect in the meta-analysis		For all 14 SAEs, there is agreement in direction of the pooled risk effect between the pairing of documents	• 16 (5 %) showed increased pooled risk of SAE on orlistat • 281 (90 %) showed no difference • 14 (5 %) showed an increased pooled risk of SAE on placebo	The one SAE showed increased pooled risk on placebo
SAE listings for when there is a change in effect including statistical significance			Two (13 %) of the 16 SAEs were statistically significant; *carotid artery stenosis, varicose veins* ^a^	One SAE; *encephalomyelitis* was statistically non-significant

Footnote:

^a^These serious adverse events were mild and unrelated to treatment

### Structured reporting of harms

The quality of reporting harms-related information, as assessed against the 15 criteria adapted from the CONSORT-harms checklist, are displayed in Table 5.

**Table 5:** Comparison of 15 harms criteria (CONSORT-harms extension used as a benchmark)

			Trial ID				
	Criteria	Description of item	NM16189	M37013	M37002	M37047	BM15421
Methods criteria	1	List addressed adverse events (AEs) with definitions.	CSR	CSR	CSR	CSR	CSR
	2	Mode of collecting harms data.	BOTH ^b^	BOTH ^b^	BOTH ^b^	CSR	BOTH ^a^
	3	Timing and time frame of surveillance for adverse events.	BOTH ^b^	Pub	CSR	NR	BOTH ^a^
	4	Attribution methods.	CSR	NR	CSR	NR	NR
	5	Intensity of ascertainment.	CSR	BOTH ^b^	CSR	CSR	CSR
	6	Harms related monitoring.	CSR	BOTH ^b^	CSR	CSR	CSR
	7	Coding of AEs.	CSR	CSR	BOTH ^a^	CSR	CSR
	8	Handling of recurrent events.	NR	CSR	NR	CSR	NR
	9	Timing issues.	CSR	CSR	CSR	NR	CSR
	10	Plans to perform any statistical analyses and inferences.	CSR	BOTH ^a^	BOTH ^a^	BOTH ^a^	BOTH ^a^
	Total items satisfied for methods criteria in clinical study report (CSR) (% of total 10 items assessed)		9 (90)	8 (80)	9 (90)	7 (70)	8 (80)
	Total items satisfied for methods criteria in publication (% of total ten items assessed)		2 (20)	5 (50)	3 (30)	1 (10)	3 (30)
Results criteria	11	Withdrawals and discontinuations.	BOTH ^a^	BOTH ^a^	BOTH ^a^	BOTH ^a^	CSR
	12	Denominators for analyses on harms.	BOTH ^b^	BOTH ^b^	BOTH ^a^	CSR	BOTH ^b^
	13	Specifying AE type.	BOTH ^a^	BOTH ^a^	BOTH ^a^	BOTH ^a^	BOTH ^a^
	14	Grading or scaling used.	NR	BOTH ^a^	BOTH ^a^	BOTH ^a^	BOTH ^a^
	15	Seriousness per arm.	BOTH ^a^	BOTH ^a^	BOTH ^a^	BOTH ^a^	BOTH ^a^
	Total items satisfied for results criteria in the CSR (% of total five items assessed)		4 (80)	5 (100)	5 (100)	5 (100)	5 (100)
	Total items satisfied for results criteria in the publication (% of total five items assessed)		4 (80)	5 (100)	5 (100)	4 (80)	4 (80)
Total items satisfied in CSR (% of total 15 items assessed)			13 (87)	13 (87)	14 (93)	12 (80)	13 (87)
Total items satisfied in publication (% of total 15 items assessed)			6 (40)	10 (67)	8 (53)	5 (33)	7 (47)

Footnote:

*BOTH* ‘reported in CSR and the corresponding journal publication’, *CSR* ‘only reported within the CSR’, *Pub* ‘only reported in journal publication’, *NR* ‘neither reported in the CSR or journals publication’. Completeness of data where agreement (BOTH) is made coded as: ^a^ ‘More complete in CSR’; ^b^ ‘Similar quality for both documents’; - ‘less complete in the CSR’

The CSRs satisfied 70–90 % the methods related criteria across the five trials compared to the journal publications, which satisfied between 10 % and 50 %. The CSRs consistently provided much greater detail regarding planned analyses than the journal publication, and on only one occasion did the journal publication provide greater detail than the CSR (trial M37013; item 3 timing and time frame of surveillance for AEs). Both the CSRs and the journal publications satisfied 80–100 % of criteria in their results sections, but greater detail was generally provided in the CSR. This included full summary tables of the AE and SAE data, including withdrawals due to harm, severity grading, and denominators for the numbers included in the safety population.

## Discussion

This case study has shown differences in the completeness and quality of reporting harms-related information between journal publications and CSRs for five orlistat trials. Information on the patient-relevant harm outcomes, including SAEs, which is required for unbiased trial evaluation, was missing from the publicly available journal article. Including these missing data from the CSRs altered the magnitude of the pooled risk difference estimates in a few cases and even resulted in five statistically significant differences (including three AEs and two SAEs). The statistically significant risk differences for AEs were faeces discolouration, dry skin, and haemorrhoids, and for SAEs, carotid artery stenosis and varicose veins. However, the statistical significance of these SAEs could not be confirmed in a sensitivity analysis pooling relative risks [27, 28] due to zero events. The events were graded mild and were classified as unrelated to treatment. Overall, the results from the journal publications in this study follow findings from past studies [15, 16], with a more detailed meta-analysis showing predominantly mild gastrointestinal harm outcomes.

The quality of reporting between journal publications and CSRs showed inconsistencies when assessed by the CONSORT-harms reporting criteria. At 70–90 %, the methods section criteria were more often satisfied in the CSRs, compared to only 10–50 % of the criteria in the journal publications. However, both document types satisfied 80–100 % of the results section criteria, albeit with greater detail being provided in the CSR. The journal publication was often incomplete when reporting planned analyses and summary tables of AEs and SAEs, which were missing information on withdrawals, severity grading, and numbers in the safety population. Journal publications are often impeded by word count restrictions. However, inadequate reporting of harms is still noticeable, even after the release of the CONSORT-harms extension [19], as the findings from our recent review [29] suggest. In contrast, CSRs have no such word restrictions imposed, and theoretically, all relevant information should be included. An alternative and more viable solution appears to be that journals should require more thorough reporting of harms via online supplements (e.g. de-identified CSRs, study protocols, and complete tables of AE-related information) [30].

In a recent study [4], findings on harms information obtained from the CSRs were found to be more complete and robust compared with the corresponding publically available sources (journal publications and registry reports). More than 86 % of all harm outcomes (AEs and SAEs) were available from the CSRs, compared to only 26 % from the journal publications. Combining harms data from registry reports and journal publications increased the proportion of outcomes to 43 %. Furthermore, withdrawals due to AEs were detailed completely in 91 % of the CSRs, with only 51 % of the journal publications providing complete information. In another study [31], inadequate reporting of the harms was shown in the Medtronic manufactured product, recombinant human bone morphogenetic protein 2 (rhBMP-2), used in spinal fusion surgery. As in our investigation, harms data were found to be missing from the publications, with considerably more data found in the corresponding trial CSRs. Further evidence of poor reporting of benefits and harms was found in a recent investigation of the product duloxetine in patients with major depressive disorder [32]. The CSRs contained extensive data on major harms that were unavailable in the journal publications and in trial registry reports. Restricting evidence synthesis to journal publications would effectively miss these important harms. Further empirical comparisons such as ours, in different clinical areas, would be valuable.

The drive to make clinical trial data more accessible has garnered widespread international support, with funders, academics, pharmaceutical industry, publishers and regulators supporting the move towards greater transparency. For example, the BMJ recently stated that it will no longer publish trials of drugs or devices where the authors do not commit to making the relevant anonymised patient-level data available; this was to be extended to all submitted clinical trials beginning 1 July 2015. In addition, the EMA has now adopted their new policy, making clinical trials data more accessible [13], including access to full CSRs. Roche should also be commended for voluntarily submitting their data and allowing further access to their CSRs. The new EU clinical trial regulation [33] published on 27 May 2014 also states under section (67) that ‘trial data should be publically accessible and presented in an easily searchable format, with related data and documents (including trial protocol and CSR) linked together by the EU trial number’.

Our study has a number of limitations. First of all, the meta-analysis results do not provide comprehensive unbiased clinical results, as they are based only on a subset of the five eligible orlistat trials, due to the inability to obtain CSRs for the remaining 26 identified trials, which were not Roche sponsored or pre-dated Roche’s policy (dating back to 1 January 1999). The meta-analyses were conducted without any adjustment for multiplicity, meaning that there is an increased chance of a false positive result, and the results should be interpreted with caution. In addition, for the five CSRs obtained from Roche in this study, some of the reports failed to include any information from modules II, III, IV, and V, and some had missing pages. Individual participant-level data and potentially other important information on harms are often presented in Roche’s CSR modules III-V. Access to these modules and confidential patient listings may have been restricted due to privacy violations, and these missing sections could present a possible cause of bias in the results. In a recent study [34], reviewers re-analysed one of SmithKline Beecham’s studies by requesting and accessing the full individual participant level data sets to compare the efficacy and safety of paroxetine. The findings from this study support the necessity of making trial individual participant-level data and protocols available to help evidence-based decisions. In module I of the CSRs, they also detailed that only commonly observed AEs (defined as those events with incidence rate in orlistat group of ≥ 5 %) were summarized, indicating that there are potentially more unreported AEs missing from the primary trial data. Therefore, the results in this study were based only on the information available.

## Conclusions

This case study confirms that CSRs can provide more complete and robust information on harms data collected in clinical trials, compared to publically available journal publications. CSRs often provide extensive information about the study methods, including design, conduct, and analysis of the trial. On the other hand, these reports are able to supplement journal publications to help facilitate the assessment of risk of bias in evidence synthesis of harm outcomes. Consequently, restricting an evidence synthesis to journal publications could have implications to systematic reviewers and other stakeholders involved in healthcare research when reaching reliable conclusions about the harmful effects of medical interventions.

## Additional files

Additional file 1: Forest plots for AEs and Search Criteria.pdf’, Figure S1: Forest plots for all adverse event MedDRA preferred terms reported in CSR and journal publications and Table S1: Search criteria used in both Cochrane central and MEDLINE. (PDF 2276 kb) Additional file 2: Reporting of AEs.xls’, Table S2 Reporting of adverse events in Clinical Study Reports (CSRs) and journal articles for Olistat trials. (XLS 31 kb) Additional file 3: Reporting of SAEs.xls’, Table S3 Reporting of serious adverse events (SAEs) in Clinical study reports (CSRs) and journal articles for Olistat trials. (XLS 65 kb) Additional file 4: SAEs Meta-Analysis results.xls’, Table S4 SAEs Meta-Analysis results. (XLS 76 kb)

## Abbreviations

AEs: adverse events
BMJ: British Medical Journal
CONSORT: Consolidated Standards of Reporting Trials
CPRD: Clinical Practice Research Data-link
CSRs: clinical study reports
EMA: European Medicines Agency
EU: European Union
JAMA: Journal of the American Medical Association
MA: meta-analysis
MedDRA: Medical Dictionary for Regulatory Activities
RAP: reporting analysis plan
RD: risk difference
RR: relative risk
SAEs: serious adverse events
Sd: standard deviation
