# Supplementary material for: Reporting of harms outcomes: a comparison of journal publications with unpublished clinical study reports of orlistat trials
Source: Trials. 2016 Apr 22;17:207. doi: 10.1186/s13063-016-1327-z (PMC4840982; doi:10.1186/s13063-016-1327-z)
Supplement: Additional file 1: — Forest plots for AEs and Search Criteria.pdf’, Figure S1: Forest plots for all adverse event MedDRA preferred terms reported in CSR and journal publications and Table S1: Search criteria used in both Cochrane central and MEDLINE. (PDF 2276 kb) [file 13063_2016_1327_MOESM1_ESM.pdf]

Reporting of harms outcomes: A comparison of journal publications with unpublished clinical study reports of orlistat trials

Alex Hodkinson[1*], Carrol Gamble[1], Catrin Tudur Smith[1]

[1]MRC North West Hub for Trials Methodology Research, Department of Biostatistics, University of Liverpool, Liverpool.

*Corresponding author
E-mail address: ahoddy@liverpool.ac.uk (A.Hodkinson).

Supplementary Figure S1: Forest plots for all adverse event MedDRA preferred terms reported in CSR and journal publications.

These meta-analysis results are based on a subset of the eligible trials of orlistat and are presented for the purpose of methodological comparison rather than definitive clinical results

Gastrointestinal disorders

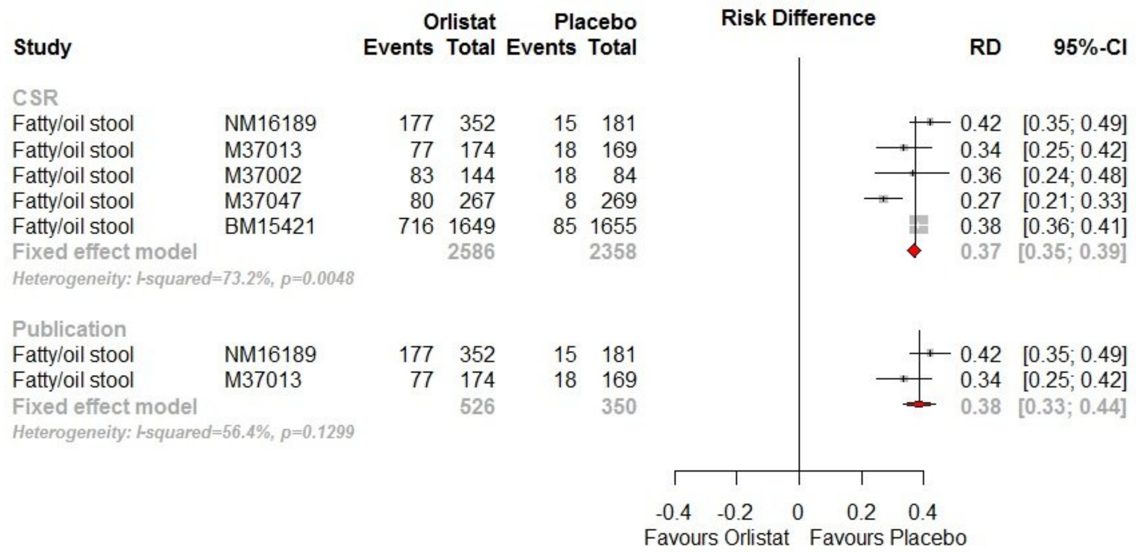

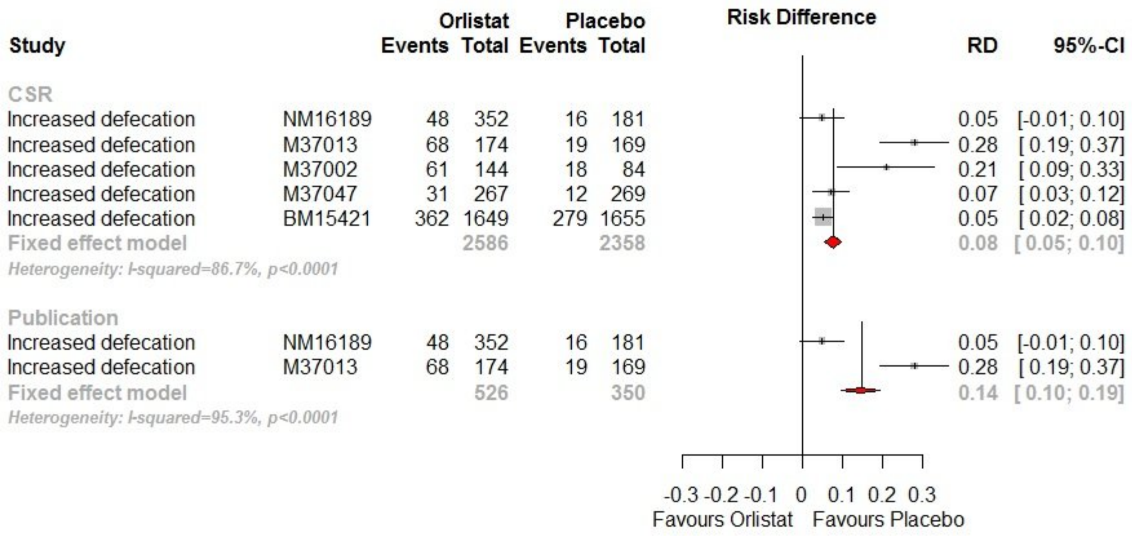
Study
Orlistat Events Total
Placebo Events Total
Risk Difference
RD 95%-CI
CSR
Increased defecation NM16189 48 352 16 181 0.05 [-0.01; 0.10]
Increased defecation M37013 68 174 19 169 0.28 [0.19; 0.37]
Increased defecation M37002 61 144 18 84 0.21 [0.09; 0.33]
Increased defecation M37047 31 267 12 269 0.07 [0.03; 0.12]
Increased defecation BM15421 362 1649 279 1655 0.05 [0.02; 0.08]
Fixed effect model 2586 2358 0.08 [0.05; 0.10]
Heterogeneity: I-squared=86.7%, p<0.0001
Publication
Increased defecation NM16189 48 352 16 181 0.05 [-0.01; 0.10]
Increased defecation M37013 68 174 19 169 0.28 [0.19; 0.37]
Fixed effect model 526 350 0.14 [0.10; 0.19]
Heterogeneity: I-squared=95.3%, p<0.0001
-0.3 -0.2 -0.1 0 0.1 0.2 0.3
Favours Orlistat Favours Placebo

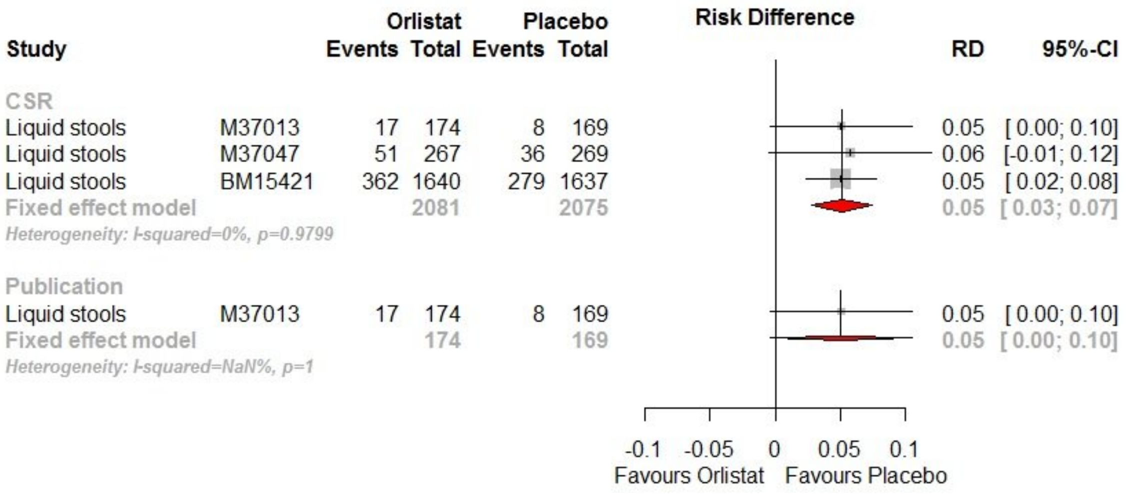
Study
Orlistat Events Total
Placebo Events Total
Risk Difference
RD 95%-CI
CSR
Liquid stools M37013 17 174 8 169 0.05 [0.00; 0.10]
Liquid stools M37047 51 267 36 269 0.06 [-0.01; 0.12]
Liquid stools BM15421 362 1640 279 1637 0.05 [0.02; 0.08]
Fixed effect model 2081 2075 0.05 [0.03; 0.07]
Heterogeneity: I-squared=0%, p=0.9799
Publication
Liquid stools M37013 17 174 8 169 0.05 [0.00; 0.10]
Fixed effect model 174 169 0.05 [0.00; 0.10]
Heterogeneity: I-squared=NaN%, p=1
-0.1 -0.05 0 0.05 0.1
Favours Orlistat Favours Placebo

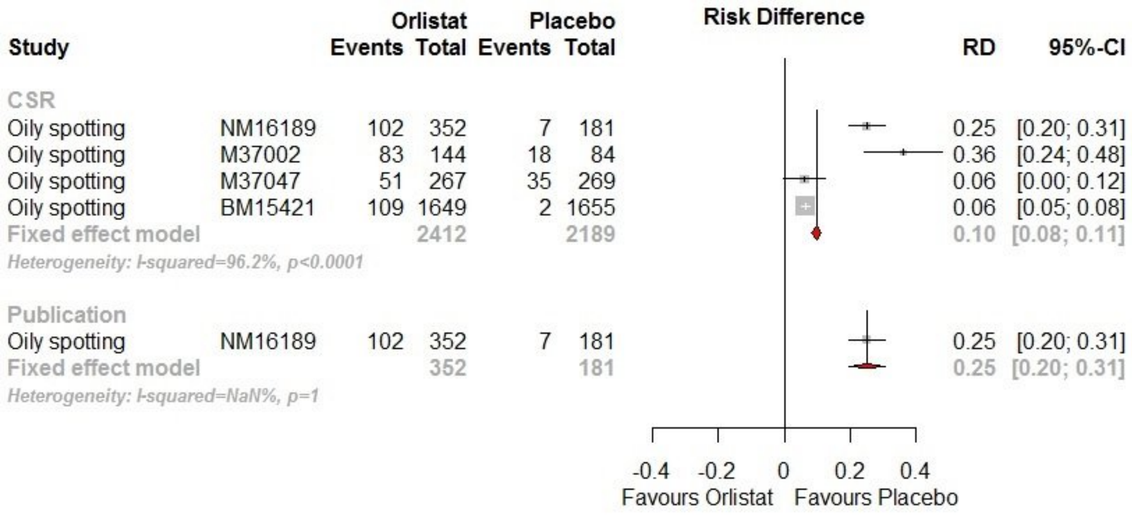
Study
Orlistat Events Total
Placebo Events Total
Risk Difference
RD 95%-CI
CSR
Oily spotting NM16189 102 352 7 181 0.25 [0.20; 0.31]
Oily spotting M37002 83 144 18 84 0.36 [0.24; 0.48]
Oily spotting M37047 51 267 35 269 0.06 [0.00; 0.12]
Oily spotting BM15421 109 1649 2 1655 0.06 [0.05; 0.08]
Fixed effect model 2412 2189 0.10 [0.08; 0.11]
Heterogeneity: I-squared=96.2%, p<0.0001
Publication
Oily spotting NM16189 102 352 7 181 0.25 [0.20; 0.31]
Fixed effect model 352 181 0.25 [0.20; 0.31]
Heterogeneity: I-squared=NaN%, p=1
-0.4 -0.2 0 0.2 0.4
Favours Orlistat Favours Placebo

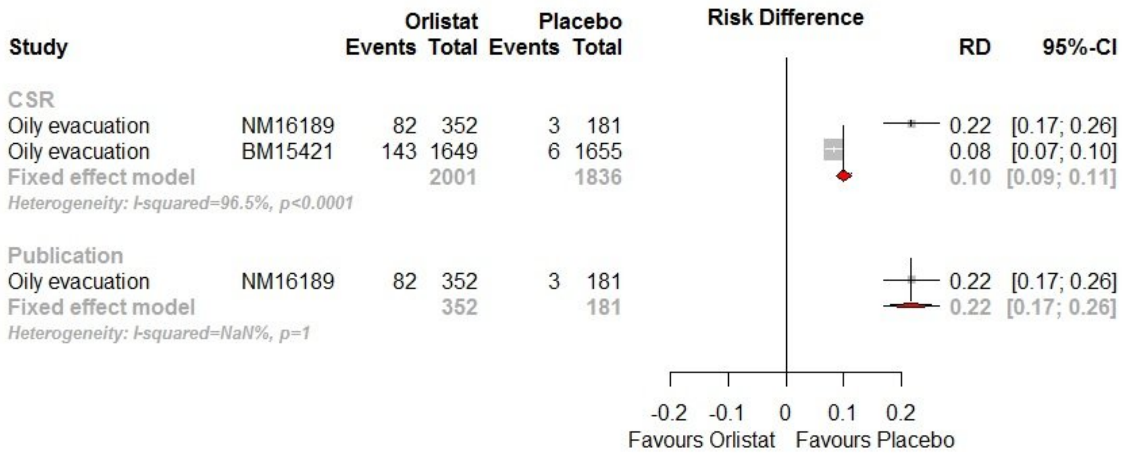
Orlistat
Placebo
Risk Difference
Study
Events Total Events Total
RD
95%-CI
CSR
Oily evacuation NM16189 82 352 3 181 0.22 [0.17; 0.26]
Oily evacuation BM15421 143 1649 6 1655 0.08 [0.07; 0.10]
Fixed effect model 2001 1836 0.10 [0.09; 0.11]
Heterogeneity: I-squared=96.5%, p<0.0001
Publication
Oily evacuation NM16189 82 352 3 181 0.22 [0.17; 0.26]
Fixed effect model 352 181 0.22 [0.17; 0.26]
Heterogeneity: I-squared=NaN%, p=1
-0.2 -0.1 0 0.1 0.2
Favours Orlistat Favours Placebo

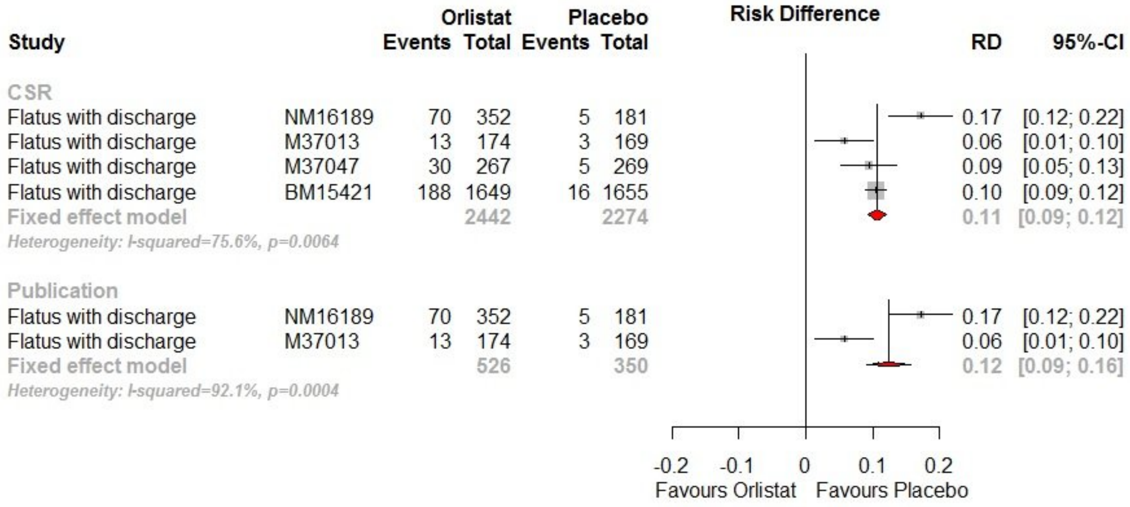
Orlistat
Placebo
Risk Difference
Study
Events Total Events Total
RD
95%-CI
CSR
Flatus with discharge NM16189 70 352 5 181 0.17 [0.12; 0.22]
Flatus with discharge M37013 13 174 3 169 0.06 [0.01; 0.10]
Flatus with discharge M37047 30 267 5 269 0.09 [0.05; 0.13]
Flatus with discharge BM15421 188 1649 16 1655 0.10 [0.09; 0.12]
Fixed effect model 2442 2274 0.11 [0.09; 0.12]
Heterogeneity: I-squared=75.6%, p=0.0064
Publication
Flatus with discharge NM16189 70 352 5 181 0.17 [0.12; 0.22]
Flatus with discharge M37013 13 174 3 169 0.06 [0.01; 0.10]
Fixed effect model 526 350 0.12 [0.09; 0.16]
Heterogeneity: I-squared=92.1%, p=0.0004
-0.2 -0.1 0 0.1 0.2
Favours Orlistat Favours Placebo

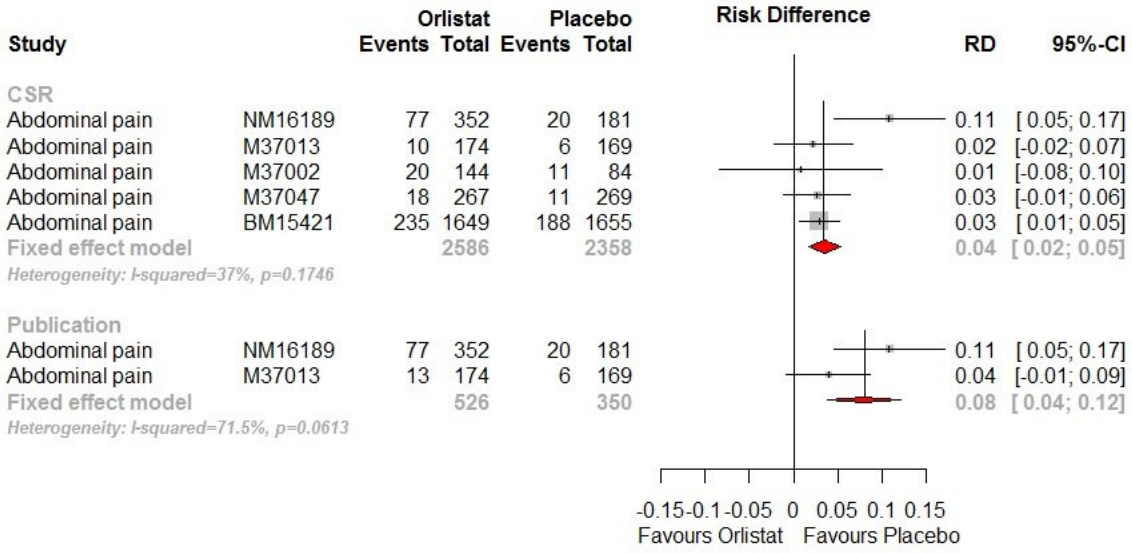
Orlistat
Placebo
Risk Difference
Study
Events Total Events Total
RD
95%-CI
CSR
Abdominal pain NM16189 77 352 20 181 0.11 [0.05; 0.17]
Abdominal pain M37013 10 174 6 169 0.02 [-0.02; 0.07]
Abdominal pain M37002 20 144 11 84 0.01 [-0.08; 0.10]
Abdominal pain M37047 18 267 11 269 0.03 [-0.01; 0.06]
Abdominal pain BM15421 235 1649 188 1655 0.03 [0.01; 0.05]
Fixed effect model 2586 2358 0.04 [0.02; 0.05]
Heterogeneity: I-squared=37%, p=0.1746
Publication
Abdominal pain NM16189 77 352 20 181 0.11 [0.05; 0.17]
Abdominal pain M37013 13 174 6 169 0.04 [-0.01; 0.09]
Fixed effect model 526 350 0.08 [0.04; 0.12]
Heterogeneity: I-squared=71.5%, p=0.0613
-0.15 -0.1 -0.05 0 0.05 0.1 0.15
Favours Orlistat Favours Placebo

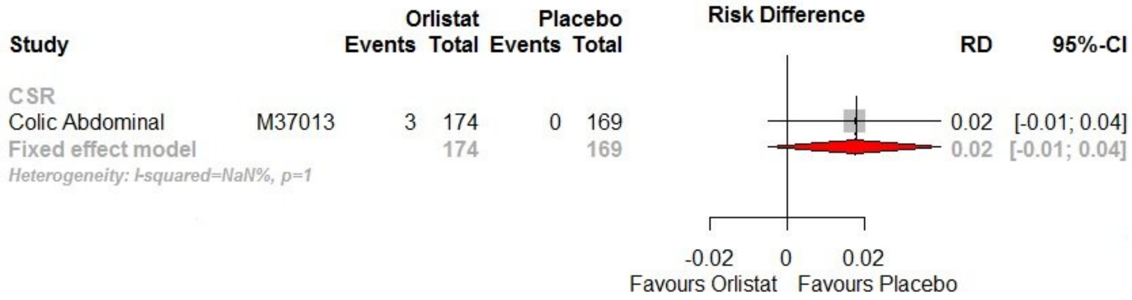
Study
Orlistat Events Total
Placebo Events Total
Risk Difference
RD 95%-CI
CSR
Colic Abdominal M37013 3 174 0 169 0.02 [-0.01; 0.04]
Fixed effect model 174 169 0.02 [-0.01; 0.04]
Heterogeneity: I-squared=NaN%, p=1
-0.02 0 0.02
Favours Orlistat Favours Placebo

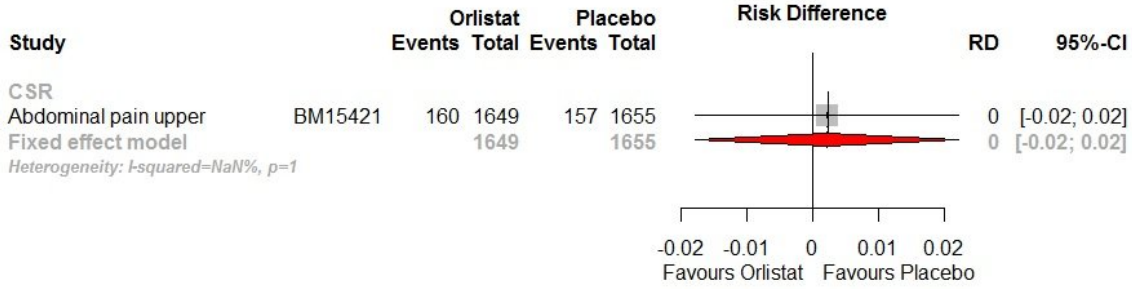
Study
Orlistat Events Total
Placebo Events Total
Risk Difference
RD 95%-CI
CSR
Abdominal pain upper BM15421 160 1649 157 1655 0 [-0.02; 0.02]
Fixed effect model 1649 1655 0 [-0.02; 0.02]
Heterogeneity: I-squared=NaN%, p=1
-0.02 -0.01 0 0.01 0.02
Favours Orlistat Favours Placebo

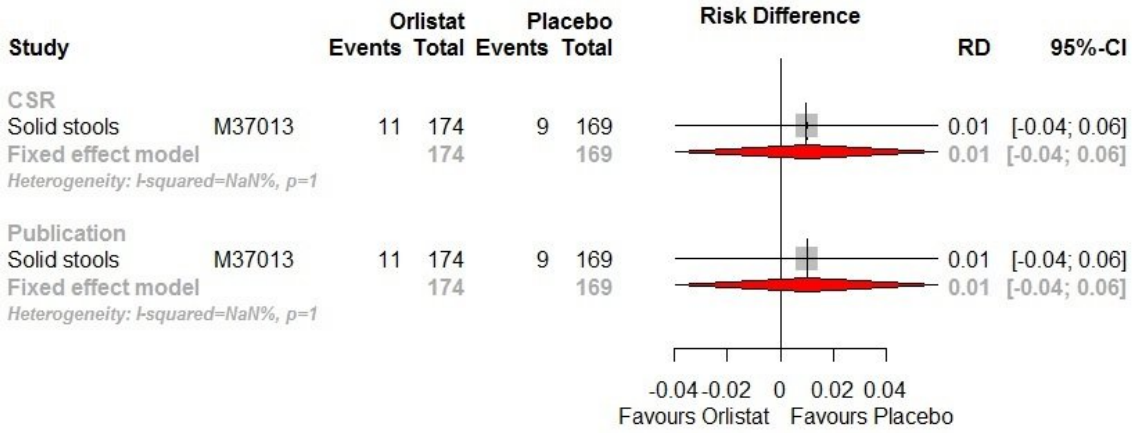
Study
Orlistat Events Total
Placebo Events Total
Risk Difference
RD 95%-CI
CSR
Solid stools M37013 11 174 9 169 0.01 [-0.04; 0.06]
Fixed effect model 174 169 0.01 [-0.04; 0.06]
Heterogeneity: I-squared=NaN%, p=1
Publication
Solid stools M37013 11 174 9 169 0.01 [-0.04; 0.06]
Fixed effect model 174 169 0.01 [-0.04; 0.06]
Heterogeneity: I-squared=NaN%, p=1
-0.04 -0.02 0 0.02 0.04
Favours Orlistat Favours Placebo

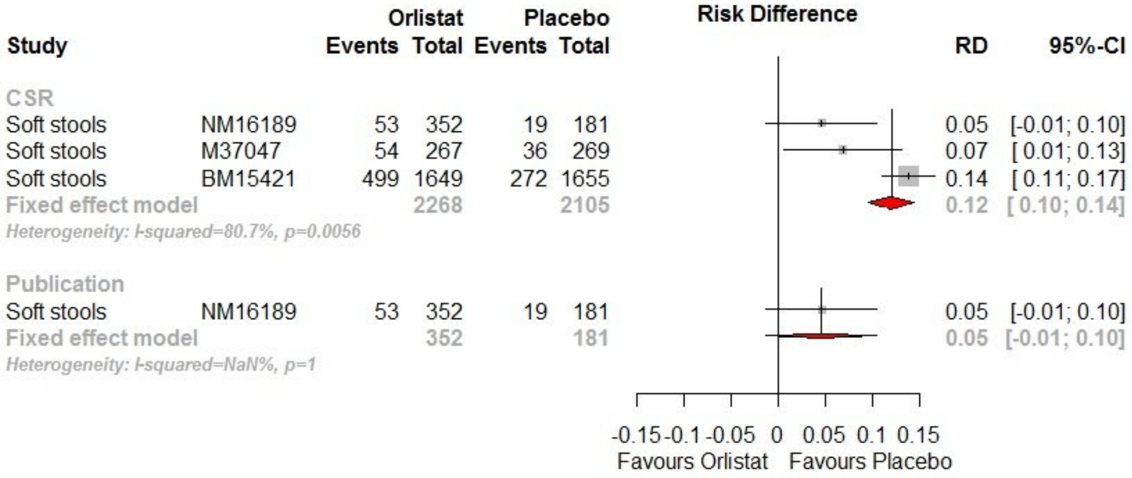
Study
Orlistat Events Total
Placebo Events Total
Risk Difference
RD 95%-CI
CSR
Soft stools NM16189 53 352 19 181 0.05 [-0.01; 0.10]
Soft stools M37047 54 267 36 269 0.07 [0.01; 0.13]
Soft stools BM15421 499 1649 272 1655 0.14 [0.11; 0.17]
Fixed effect model 2268 2105 0.12 [0.10; 0.14]
Heterogeneity: I-squared=80.7%, p=0.0056
Publication
Soft stools NM16189 53 352 19 181 0.05 [-0.01; 0.10]
Fixed effect model 352 181 0.05 [-0.01; 0.10]
Heterogeneity: I-squared=NaN%, p=1
-0.15 -0.1 -0.05 0 0.05 0.1 0.15
Favours Orlistat Favours Placebo

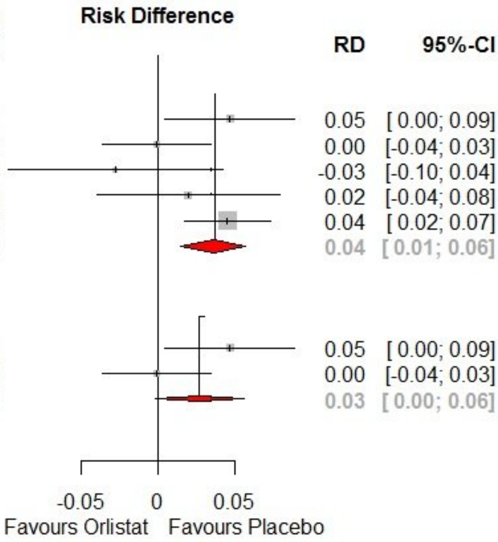

Study		Orlistat Events	Orlistat Total	Placebo Events	Placebo Total	RD	95%-CI
CSR							
Flatulence	NM16189	32	352	8	181	0.05	[0.00; 0.09]
Flatulence	M37013	5	174	5	169	0.00	[-0.04; 0.03]
Flatulence	M37002	8	144	7	84	-0.03	[-0.10; 0.04]
Flatulence	M37047	41	267	36	269	0.02	[-0.04; 0.08]
Flatulence	BM15421	400	1649	327	1655	0.04	[0.02; 0.07]
Fixed effect model			2586		2358	0.04	[0.01; 0.06]
Heterogeneity: I-squared=51.4%, p=0.0837							
Publication							
Flatulence	NM16189	32	352	8	181	0.05	[0.00; 0.09]
Flatulence	M37013	5	174	5	169	0.00	[-0.04; 0.03]
Fixed effect model			526		350	0.03	[0.00; 0.06]
Heterogeneity: I-squared=68.4%, p=0.0752							

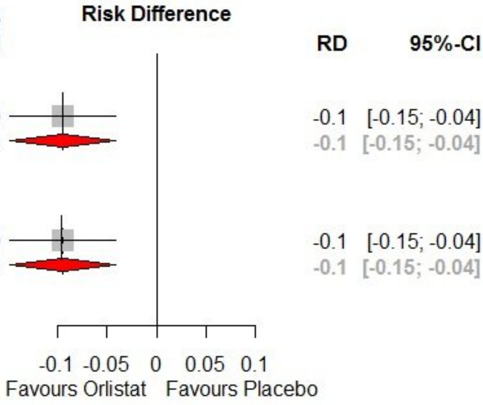

Study		Orlistat Events	Orlistat Total	Placebo Events	Placebo Total	RD	95%-CI
CSR							
Decreased defecation	M37013	4	174	20	169	-0.1	[-0.15; -0.04]
Fixed effect model			174		169	-0.1	[-0.15; -0.04]
Heterogeneity: I-squared=NaN%, p=1							
Publication							
Decreased defecation	M37013	4	174	20	169	-0.1	[-0.15; -0.04]
Fixed effect model			174		169	-0.1	[-0.15; -0.04]
Heterogeneity: I-squared=NaN%, p=1							

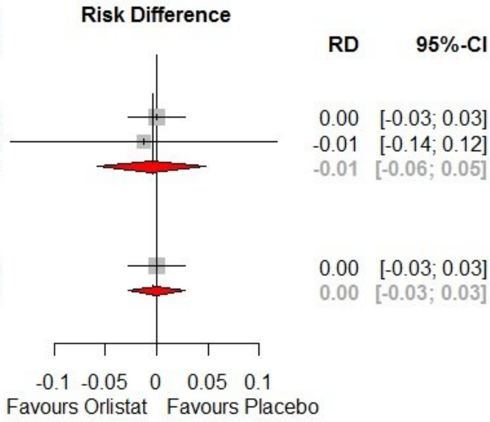

Study		Orlistat Events	Orlistat Total	Placebo Events	Placebo Total	RD	95%-CI
CSR							
Abdominal distension	M37013	3	174	3	169	0.00	[-0.03; 0.03]
Abdominal distension	M37002	53	144	32	84	-0.01	[-0.14; 0.12]
Fixed effect model			318		253	-0.01	[-0.06; 0.05]
Heterogeneity: I-squared=0%, p=0.7236							
Publication							
Abdominal distension	M37013	3	174	3	169	0.00	[-0.03; 0.03]
Fixed effect model			174		169	0.00	[-0.03; 0.03]
Heterogeneity: I-squared=NaN%, p=1							

Study		Orlistat Events	Orlistat Total	Placebo Events	Placebo Total	RD	95%-CI
**CSR**							
Faecal incontinence	NM16189	31	352	1	181	0.08	[0.05; 0.11]
Faecal incontinence	M37047	26	267	6	269	0.08	[0.04; 0.11]
Faecal incontinence	BM15421	60	1649	4	1655	0.03	[0.02; 0.04]
**Fixed effect model**			2268		2105	**0.04**	**[0.04; 0.05]**
Heterogeneity: I-squared=84.3%, p=0.0017							
**Publication**							
Faecal incontinence	NM16189	31	352	1	181	0.08	[0.05; 0.11]
**Fixed effect model**			352		181	**0.08**	**[0.05; 0.11]**
Heterogeneity: I-squared=NaN%, p=1							

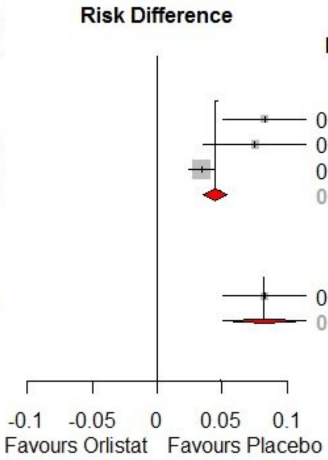

Study		Orlistat Events	Orlistat Total	Placebo Events	Placebo Total	RD	95%-CI
**CSR**							
Faecal urgency	NM16189	73	352	20	181	0.10	[0.03; 0.16]
Faecal urgency	M37013	2	174	4	169	-0.01	[-0.04; 0.02]
Faecal urgency	M37002	73	144	19	84	0.28	[0.16; 0.40]
Faecal urgency	M37047	25	267	8	269	0.06	[0.02; 0.10]
Faecal urgency	BM15421	301	1649	87	1655	0.13	[0.11; 0.15]
**Fixed effect model**			2586		2358	**0.12**	**[0.10; 0.13]**
Heterogeneity: I-squared=95.9%, p<0.0001							
**Publication**							
Faecal urgency	NM16189	73	352	20	181	0.10	[0.03; 0.16]
Faecal urgency	M37013	2	174	4	169	-0.01	[-0.04; 0.02]
**Fixed effect model**			526		350	**0.05**	**[0.01; 0.09]**
Heterogeneity: I-squared=95.5%, p<0.0001							

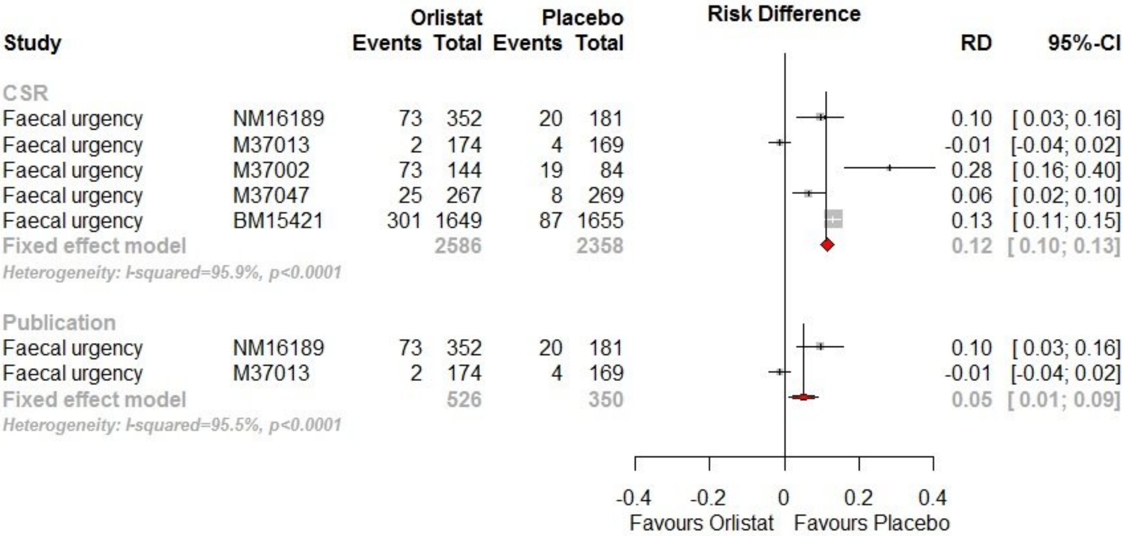

Study		Orlistat Events	Orlistat Total	Placebo Events	Placebo Total	RD	95%-CI
**CSR**							
Faeces discoloured	NM16189	16	352	3	181	0.03	[0.00; 0.06]
Faeces discoloured	M37047	9	267	8	269	0.00	[-0.03; 0.03]
Faeces discoloured	BM15421	40	1649	4	1655	0.02	[0.01; 0.03]
**Fixed effect model**			2268		2105	**0.02**	**[0.01; 0.03]**
Heterogeneity: I-squared=0%, p=0.4391							

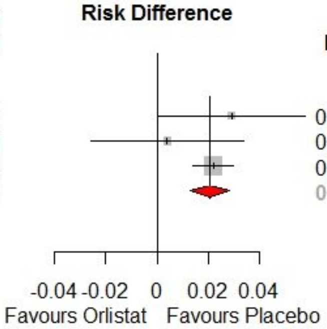

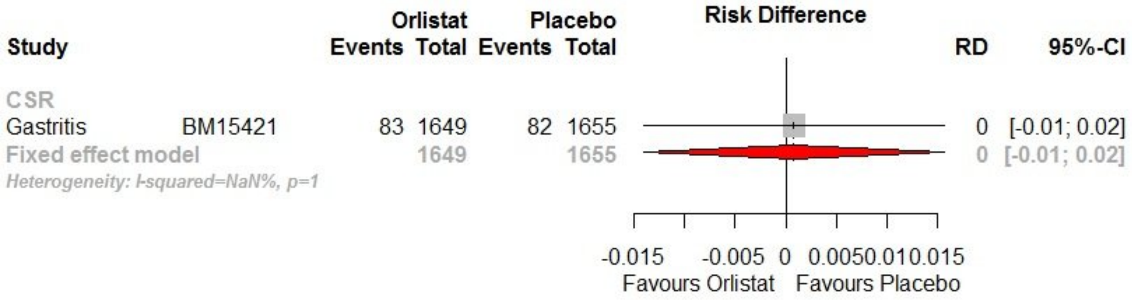
Study
Orlistat Events Total
Placebo Events Total
Risk Difference
RD 95%-CI
CSR
Gastritis BM15421 83 1649 82 1655 0 [-0.01; 0.02]
Fixed effect model 1649 1655 0 [-0.01; 0.02]
Heterogeneity: I-squared=NaN%, p=1
-0.015 -0.005 0 0.0050.010.015
Favours Orlistat Favours Placebo

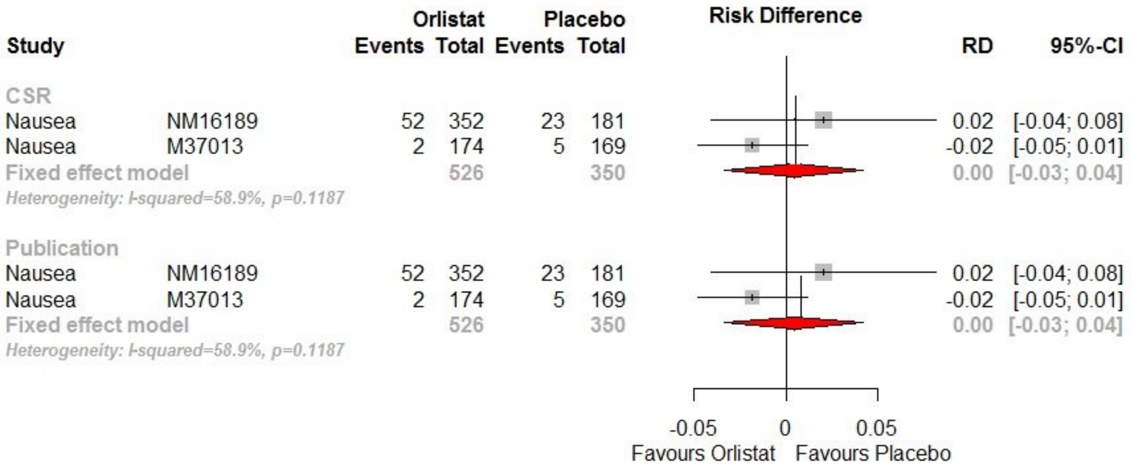
Study
Orlistat Events Total
Placebo Events Total
Risk Difference
RD 95%-CI
CSR
Nausea NM16189 52 352 23 181 0.02 [-0.04; 0.08]
Nausea M37013 2 174 5 169 -0.02 [-0.05; 0.01]
Fixed effect model 526 350 0.00 [-0.03; 0.04]
Heterogeneity: I-squared=58.9%, p=0.1187
Publication
Nausea NM16189 52 352 23 181 0.02 [-0.04; 0.08]
Nausea M37013 2 174 5 169 -0.02 [-0.05; 0.01]
Fixed effect model 526 350 0.00 [-0.03; 0.04]
Heterogeneity: I-squared=58.9%, p=0.1187
-0.05 0 0.05
Favours Orlistat Favours Placebo

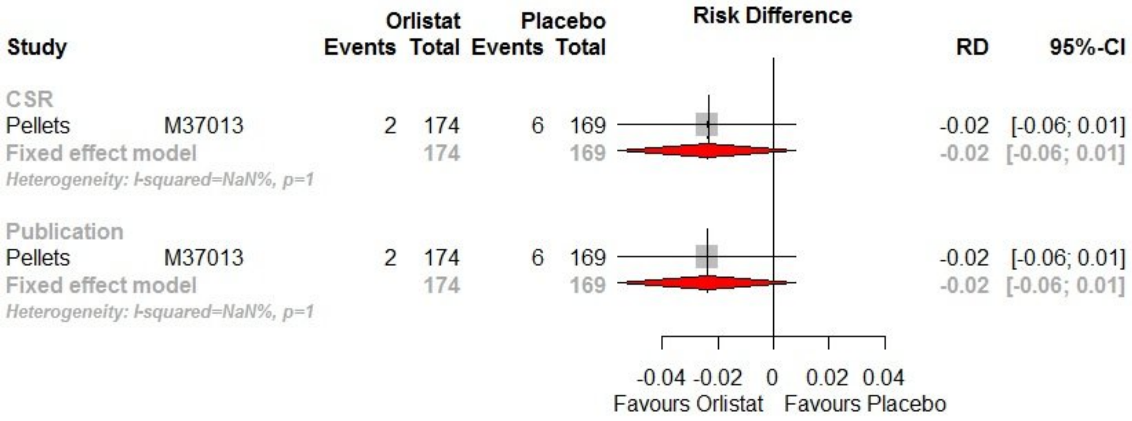
Study
Orlistat Events Total
Placebo Events Total
Risk Difference
RD 95%-CI
CSR
Pellets M37013 2 174 6 169 -0.02 [-0.06; 0.01]
Fixed effect model 174 169 -0.02 [-0.06; 0.01]
Heterogeneity: I-squared=NaN%, p=1
Publication
Pellets M37013 2 174 6 169 -0.02 [-0.06; 0.01]
Fixed effect model 174 169 -0.02 [-0.06; 0.01]
Heterogeneity: I-squared=NaN%, p=1
-0.04 -0.02 0 0.02 0.04
Favours Orlistat Favours Placebo

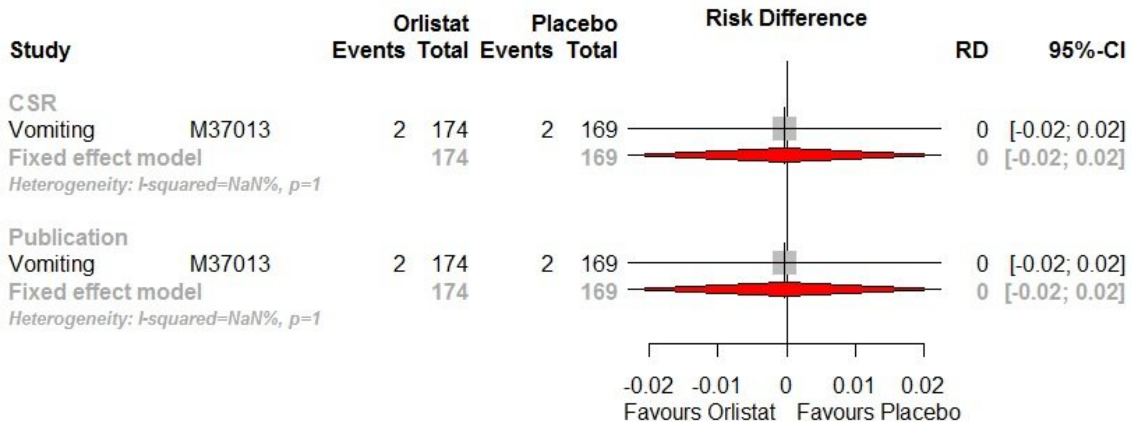
Study
Orlistat Events Total
Placebo Events Total
Risk Difference
RD 95%-CI
CSR
Vomiting M37013 2 174 2 169 0 [-0.02; 0.02]
Fixed effect model 174 169 0 [-0.02; 0.02]
Heterogeneity: I-squared=NaN%, p=1
Publication
Vomiting M37013 2 174 2 169 0 [-0.02; 0.02]
Fixed effect model 174 169 0 [-0.02; 0.02]
Heterogeneity: I-squared=NaN%, p=1
-0.02 -0.01 0 0.01 0.02
Favours Orlistat Favours Placebo

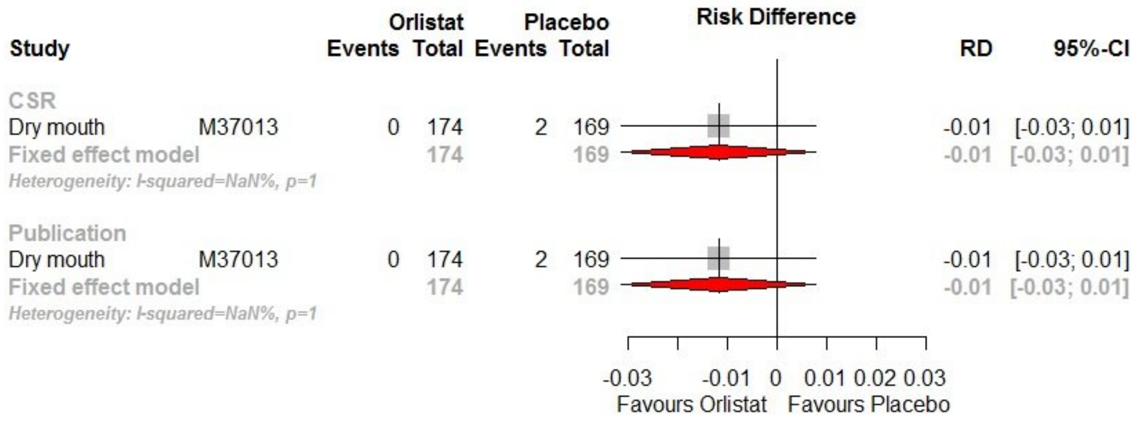
Study
Orlistat Events Total
Placebo Events Total
Risk Difference
RD 95%-CI
CSR
Dry mouth M37013 0 174 2 169 -0.01 [-0.03; 0.01]
Fixed effect model 174 169 -0.01 [-0.03; 0.01]
Heterogeneity: I-squared=NaN%, p=1
Publication
Dry mouth M37013 0 174 2 169 -0.01 [-0.03; 0.01]
Fixed effect model 174 169 -0.01 [-0.03; 0.01]
Heterogeneity: I-squared=NaN%, p=1
-0.03 -0.01 0 0.01 0.02 0.03
Favours Orlistat Favours Placebo

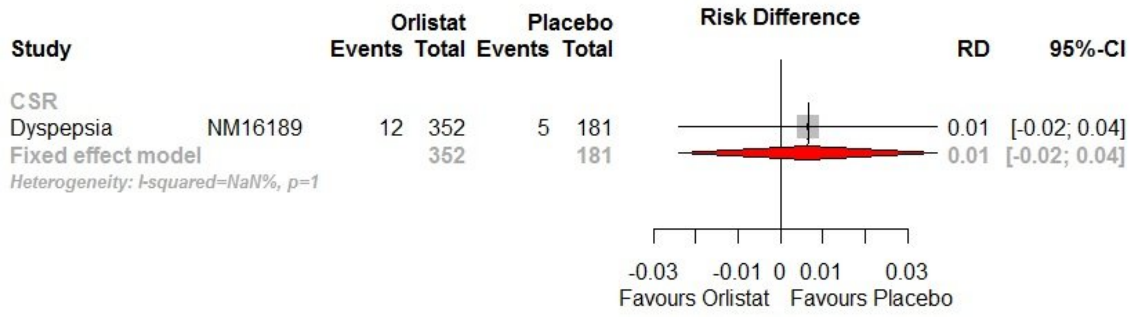
Study
Orlistat Events Total
Placebo Events Total
Risk Difference
RD 95%-CI
CSR
Dyspepsia NM16189 12 352 5 181 0.01 [-0.02; 0.04]
Fixed effect model 352 181 0.01 [-0.02; 0.04]
Heterogeneity: I-squared=NaN%, p=1
-0.03 -0.01 0 0.01 0.03
Favours Orlistat Favours Placebo

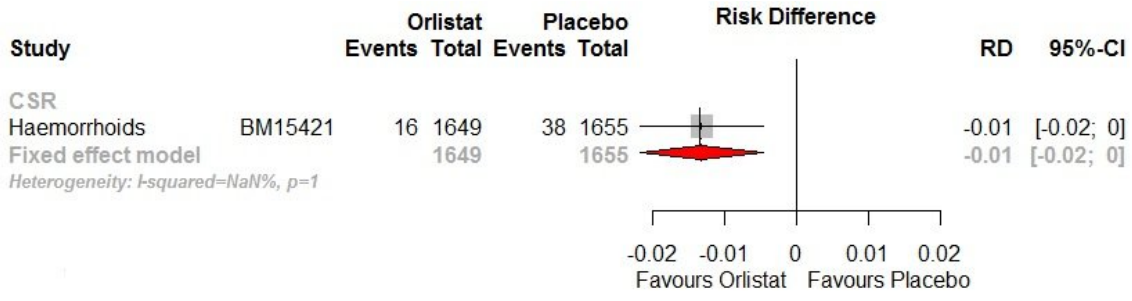
Study
Orlistat Events Total
Placebo Events Total
Risk Difference
RD 95%-CI
CSR
Haemorrhoids BM15421 16 1649 38 1655 -0.01 [-0.02; 0]
Fixed effect model 1649 1655 -0.01 [-0.02; 0]
Heterogeneity: I-squared=NaN%, p=1
-0.02 -0.01 0 0.01 0.02
Favours Orlistat Favours Placebo

Infections and infestations

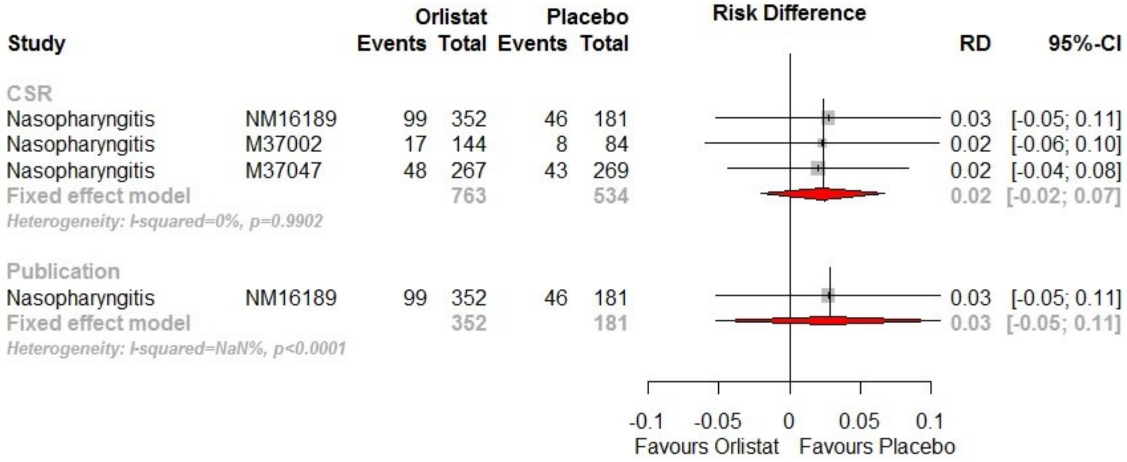

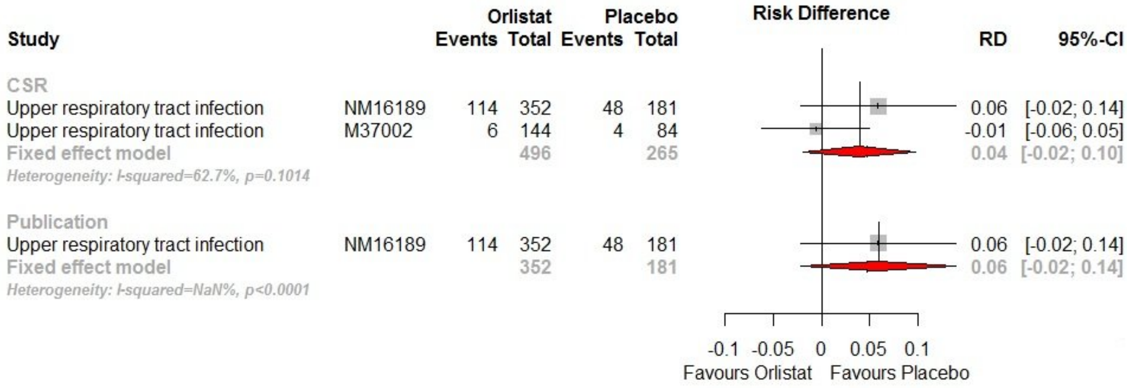

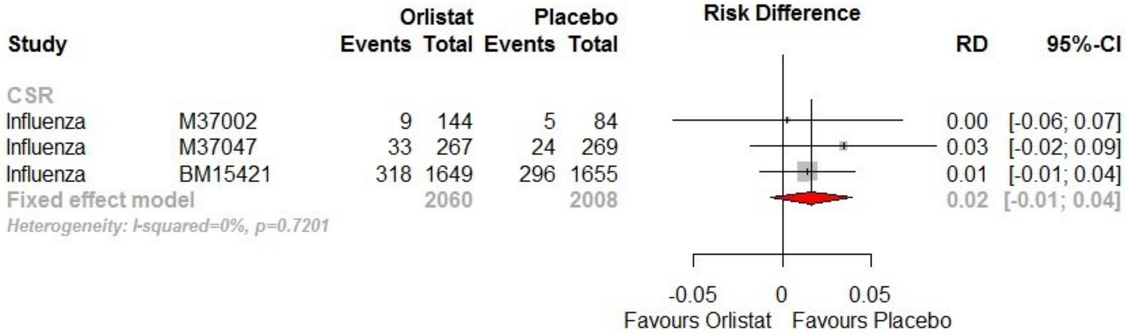

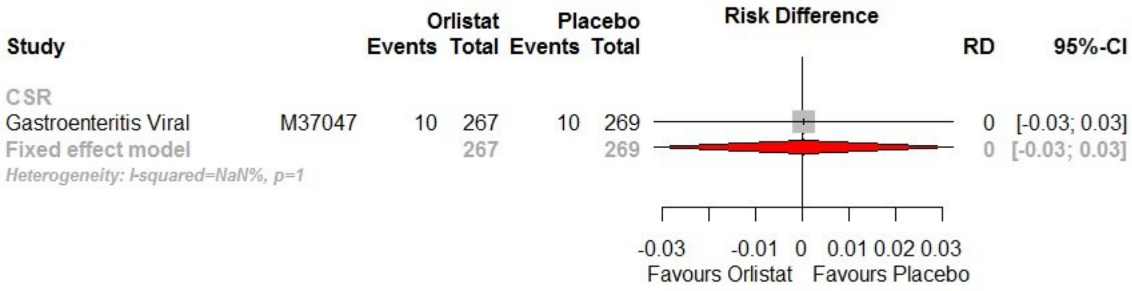
Study
Orlistat
Events Total
Placebo
Events Total
Risk Difference
RD
95%-CI
CSR
Gastroenteritis Viral M37047 10 267 10 269 0 [-0.03; 0.03]
Fixed effect model 267 269 0 [-0.03; 0.03]
Heterogeneity: I-squared=NaN%, p=1
-0.03 -0.01 0 0.01 0.02 0.03
Favours Orlistat
Favours Placebo

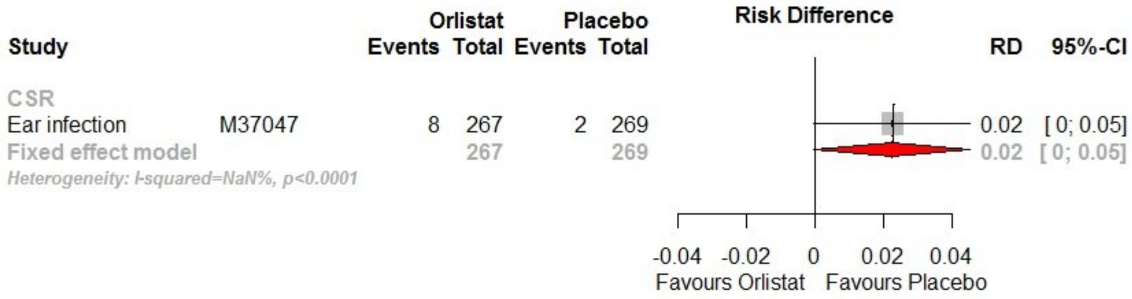
Study
Orlistat
Events Total
Placebo
Events Total
Risk Difference
RD
95%-CI
CSR
Ear infection M37047 8 267 2 269 0.02 [0; 0.05]
Fixed effect model 267 269 0.02 [0; 0.05]
Heterogeneity: I-squared=NaN%, p<0.0001
-0.04 -0.02 0 0.02 0.04
Favours Orlistat
Favours Placebo

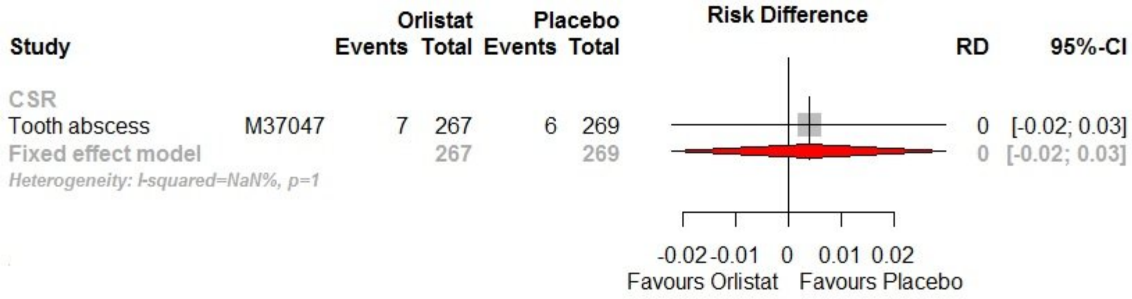
Study
Orlistat
Events Total
Placebo
Events Total
Risk Difference
RD
95%-CI
CSR
Tooth abscess M37047 7 267 6 269 0 [-0.02; 0.03]
Fixed effect model 267 269 0 [-0.02; 0.03]
Heterogeneity: I-squared=NaN%, p=1
-0.02 -0.01 0 0.01 0.02
Favours Orlistat
Favours Placebo

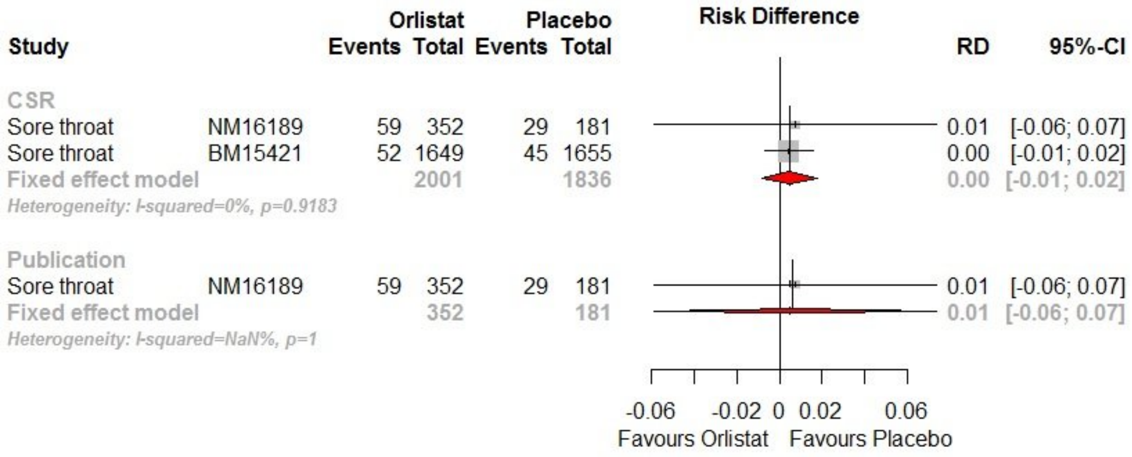
Study
Orlistat
Events Total
Placebo
Events Total
Risk Difference
RD
95%-CI
CSR
Sore throat NM16189 59 352 29 181 0.01 [-0.06; 0.07]
Sore throat BM15421 52 1649 45 1655 0.00 [-0.01; 0.02]
Fixed effect model 2001 1836 0.00 [-0.01; 0.02]
Heterogeneity: I-squared=0%, p=0.9183
Publication
Sore throat NM16189 59 352 29 181 0.01 [-0.06; 0.07]
Fixed effect model 352 181 0.01 [-0.06; 0.07]
Heterogeneity: I-squared=NaN%, p=1
-0.06 -0.02 0 0.02 0.06
Favours Orlistat
Favours Placebo

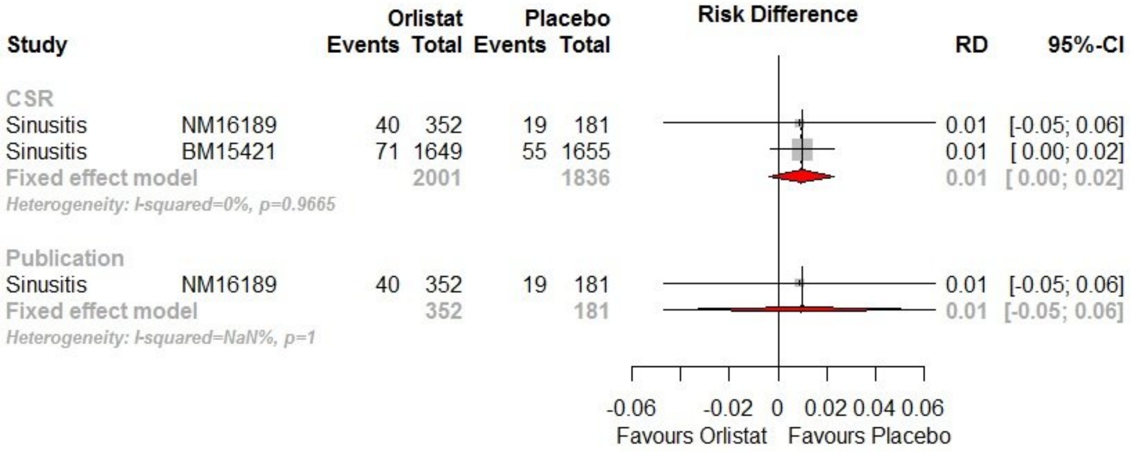
Study
Orlistat Events Total
Placebo Events Total
Risk Difference
RD 95%-CI
CSR
Sinusitis NM16189 40 352 19 181 0.01 [-0.05; 0.06]
Sinusitis BM15421 71 1649 55 1655 0.01 [0.00; 0.02]
Fixed effect model 2001 1836 0.01 [0.00; 0.02]
Heterogeneity: I-squared=0%, p=0.9665
Publication
Sinusitis NM16189 40 352 19 181 0.01 [-0.05; 0.06]
Fixed effect model 352 181 0.01 [-0.05; 0.06]
Heterogeneity: I-squared=NaN%, p=1
-0.06 -0.02 0 0.02 0.04 0.06
Favours Orlistat Favours Placebo

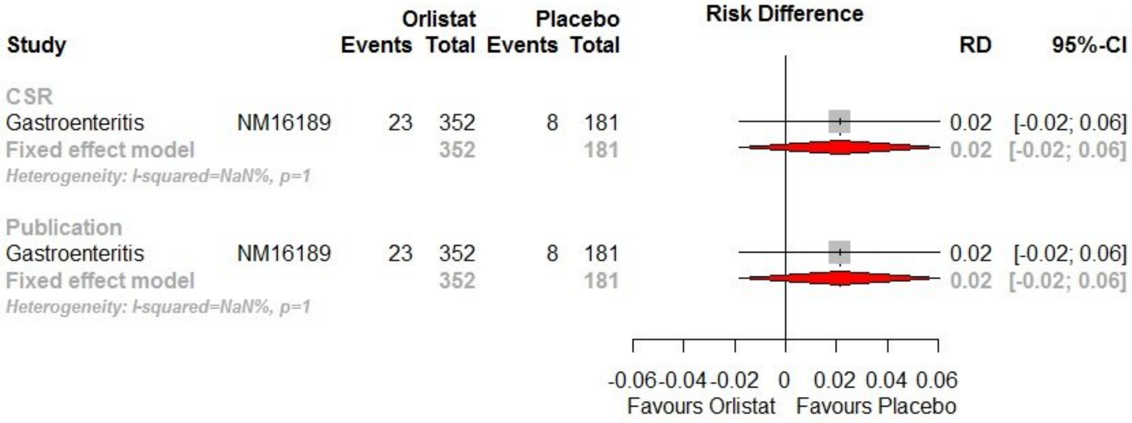
Study
Orlistat Events Total
Placebo Events Total
Risk Difference
RD 95%-CI
CSR
Gastroenteritis NM16189 23 352 8 181 0.02 [-0.02; 0.06]
Fixed effect model 352 181 0.02 [-0.02; 0.06]
Heterogeneity: I-squared=NaN%, p=1
Publication
Gastroenteritis NM16189 23 352 8 181 0.02 [-0.02; 0.06]
Fixed effect model 352 181 0.02 [-0.02; 0.06]
Heterogeneity: I-squared=NaN%, p=1
-0.06 -0.04 -0.02 0 0.02 0.04 0.06
Favours Orlistat Favours Placebo

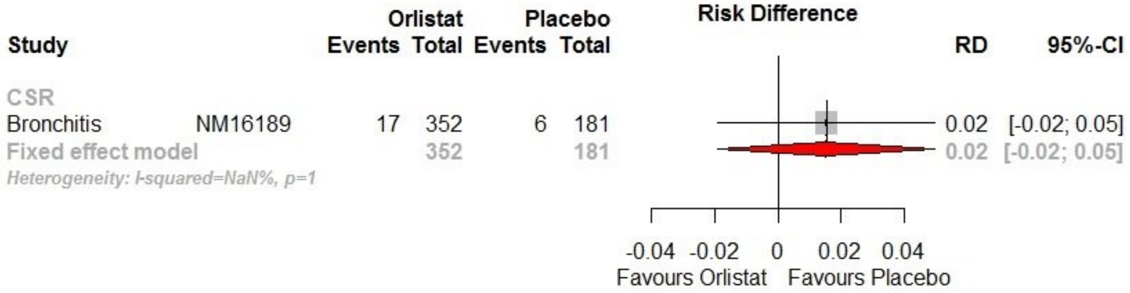
Study
Orlistat Events Total
Placebo Events Total
Risk Difference
RD 95%-CI
CSR
Bronchitis NM16189 17 352 6 181 0.02 [-0.02; 0.05]
Fixed effect model 352 181 0.02 [-0.02; 0.05]
Heterogeneity: I-squared=NaN%, p=1
-0.04 -0.02 0 0.02 0.04
Favours Orlistat Favours Placebo

Study		Orlistat Events	Orlistat Total	Placebo Events	Placebo Total	RD	95%-CI
CSR							
Pharyngitis	NM16189	11	352	4	181	0.01	[-0.02; 0.04]
Fixed effect model			352		181	0.01	[-0.02; 0.04]
Heterogeneity: I-squared=NaN%, p=1							

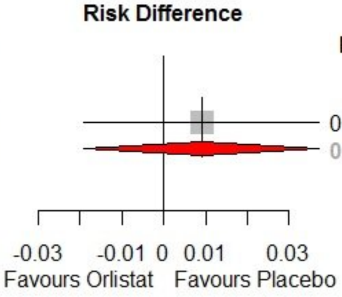

Study		Orlistat Events	Orlistat Total	Placebo Events	Placebo Total	RD	95%-CI
CSR							
Viral infection	NM16189	10	352	3	181	0.01	[-0.01; 0.04]
Fixed effect model			352		181	0.01	[-0.01; 0.04]
Heterogeneity: I-squared=NaN%, p=1							

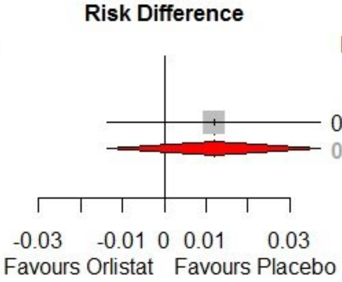

Study		Orlistat Events	Orlistat Total	Placebo Events	Placebo Total	RD	95%-CI
CSR							
Upper respiratory tract infection viral	NM16189	8	352	1	181	0.02	[0; 0.04]
Fixed effect model			352		181	0.02	[0; 0.04]
Heterogeneity: I-squared=NaN%, p=1							

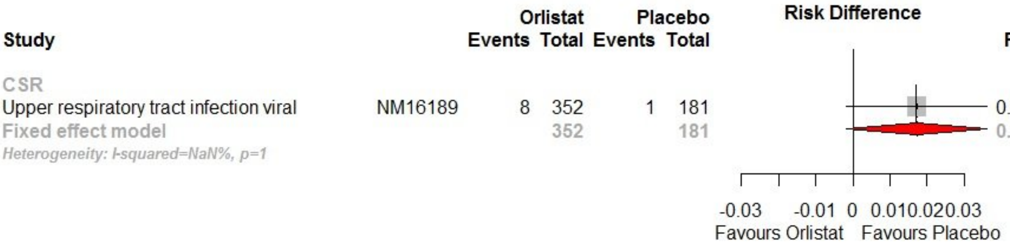

Study		Orlistat Events	Orlistat Total	Placebo Events	Placebo Total	RD	95%-CI
CSR							
Urinary tract infection	M37002	11	144	8	84	-0.02	[-0.1; 0.06]
Fixed effect model			144		84	-0.02	[-0.1; 0.06]
Heterogeneity: I-squared=NaN%, p=1							

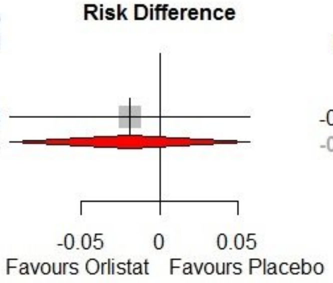

Metabolism and nutrition disorders

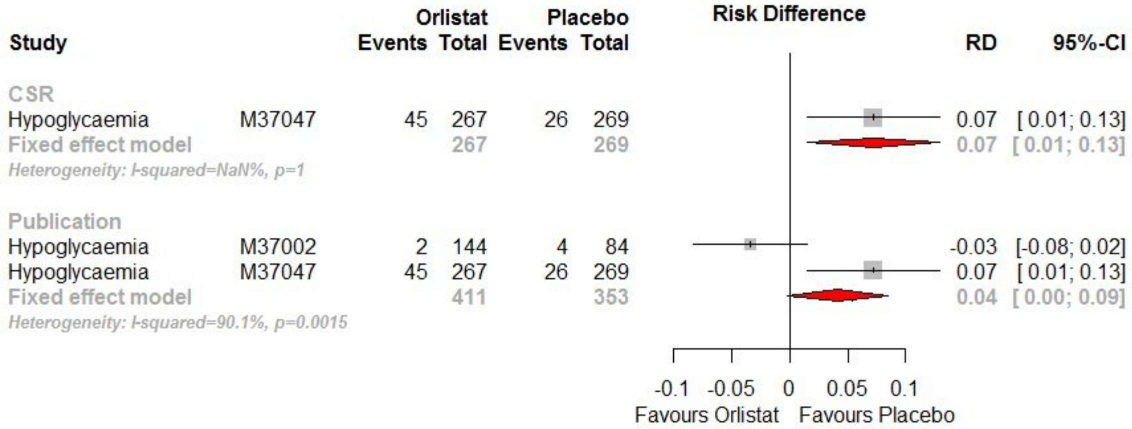

General disorders and administration site conditions

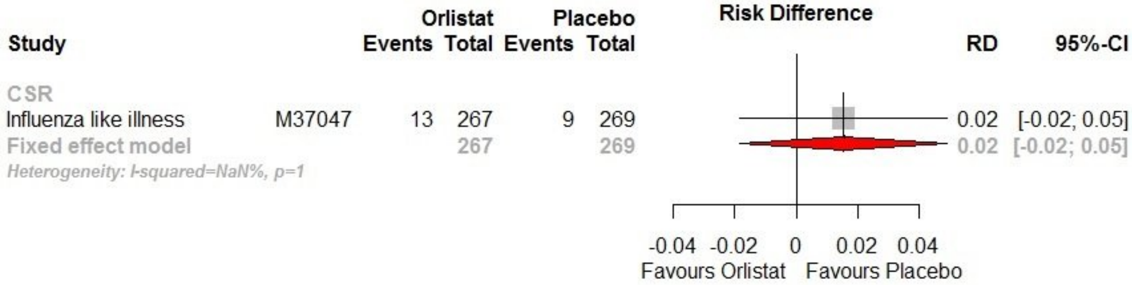

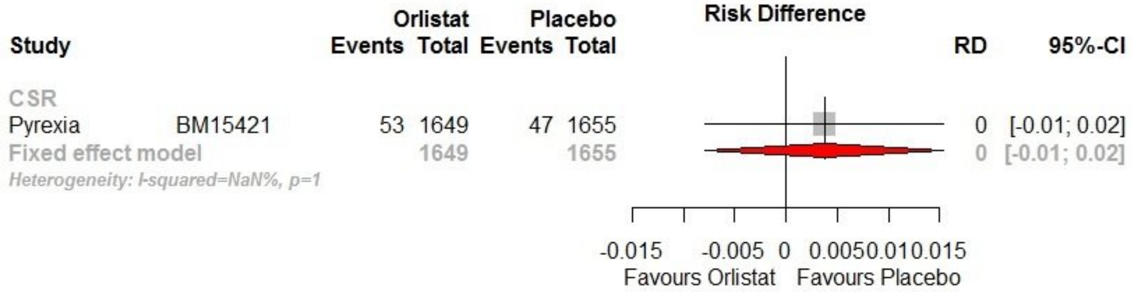

Nervous system disorders

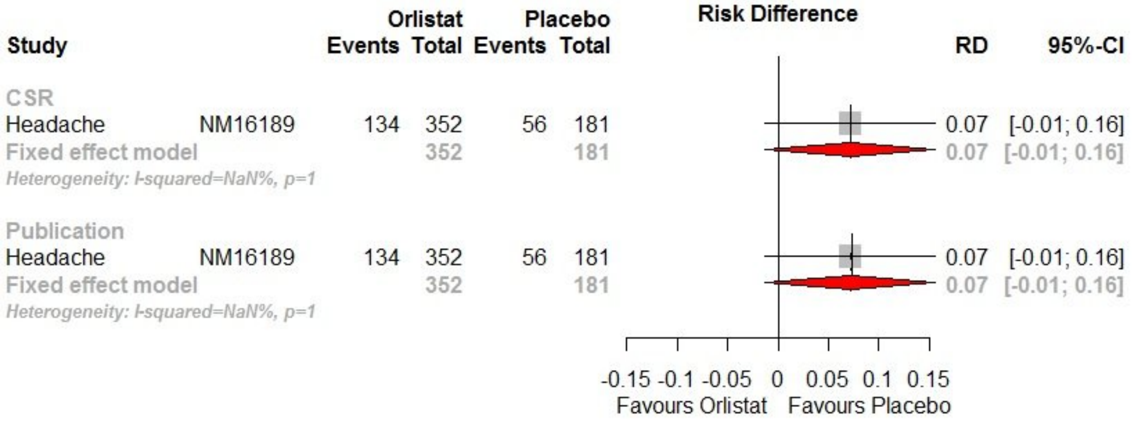

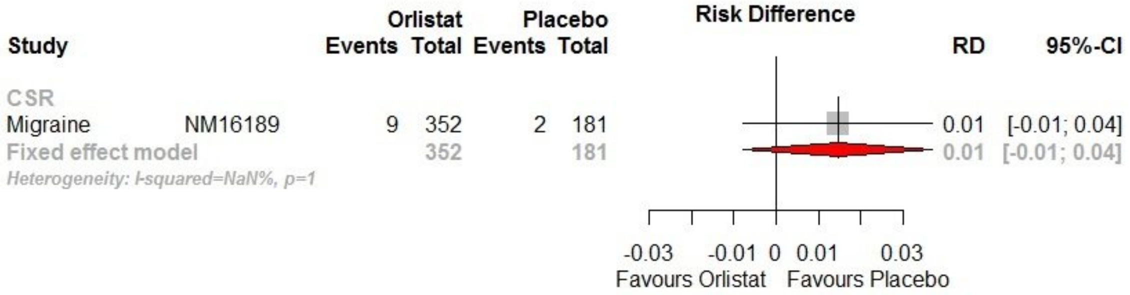

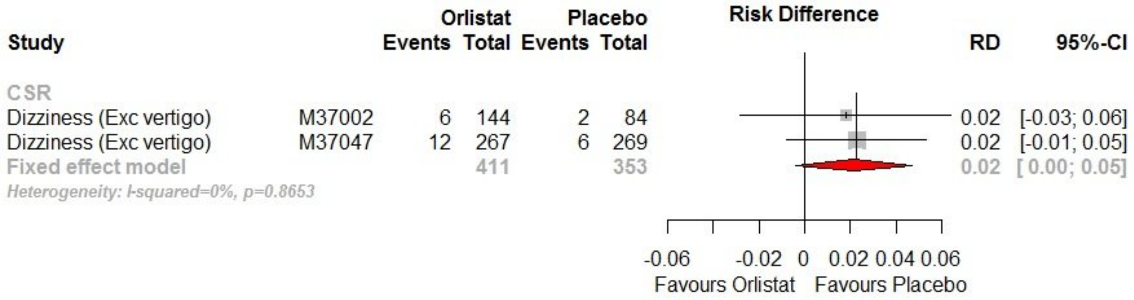

Psychiatric disorders

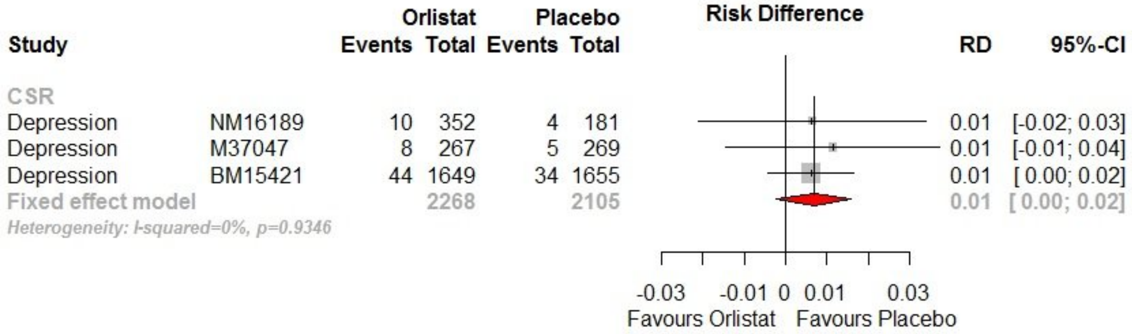

Respiratory, thoracic and medicinal disorders

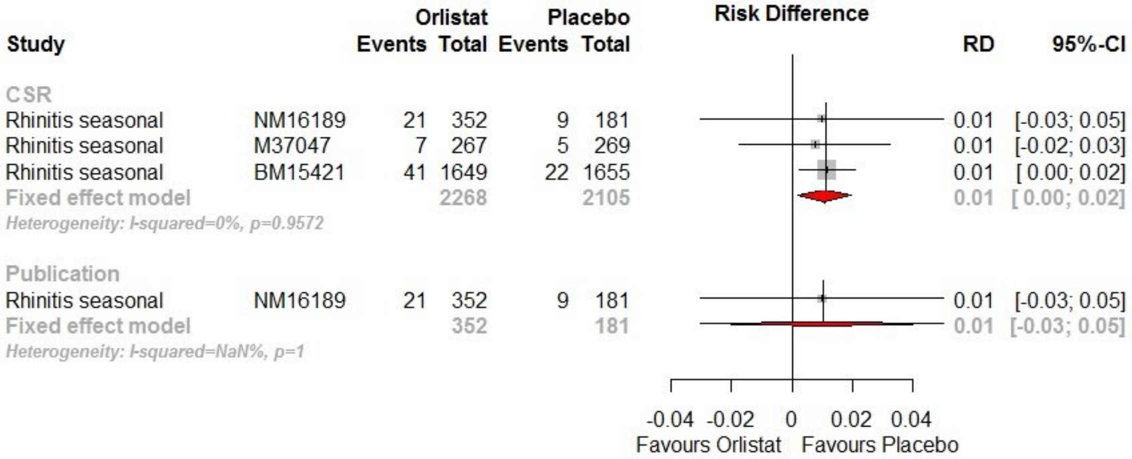

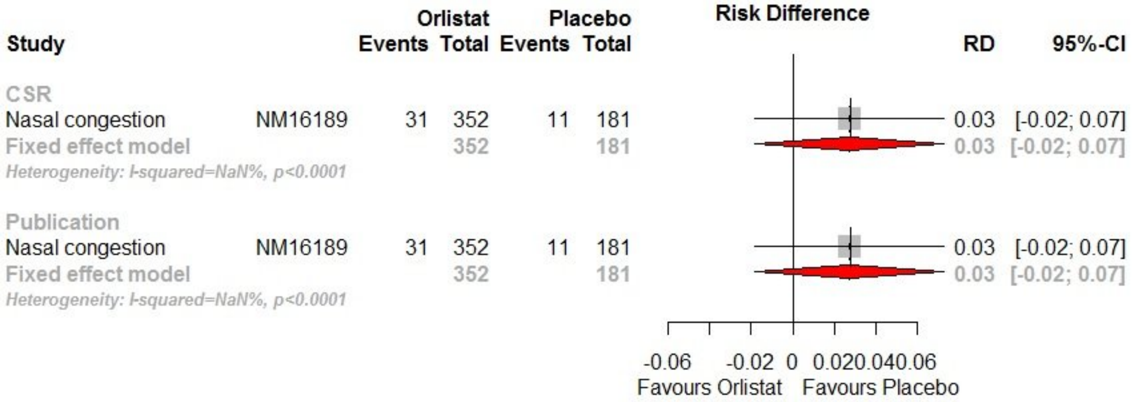

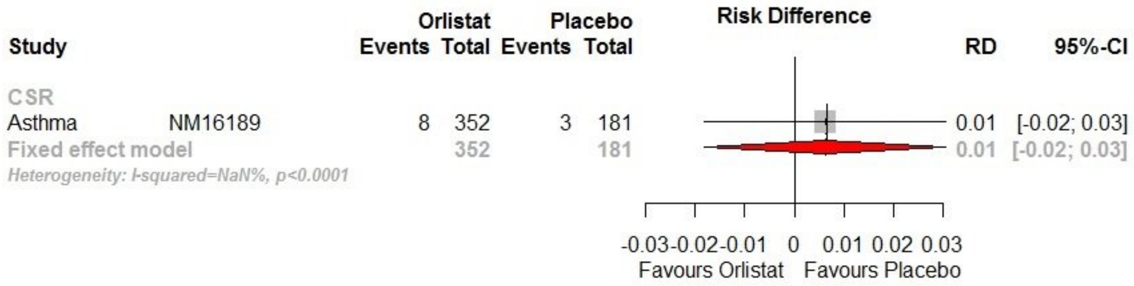

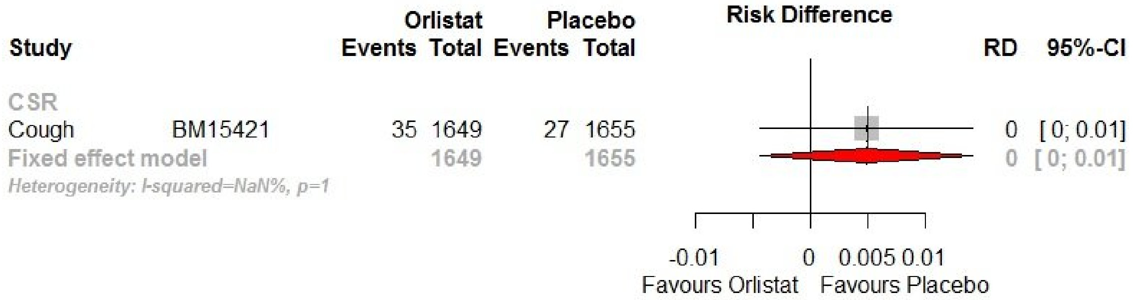

Skin & Subcutaneous tissue disorders

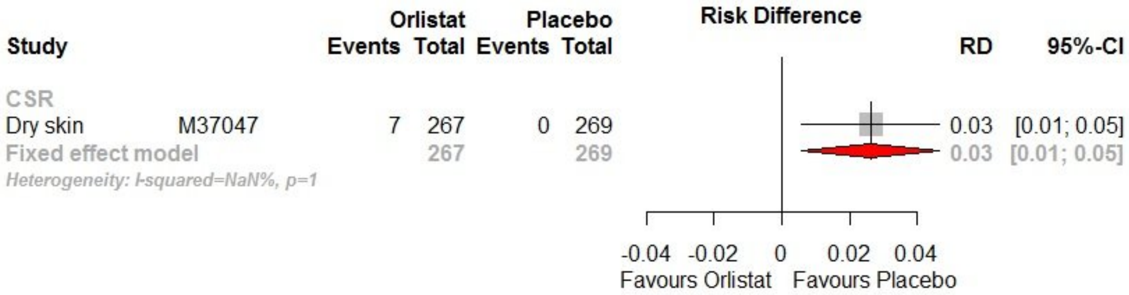

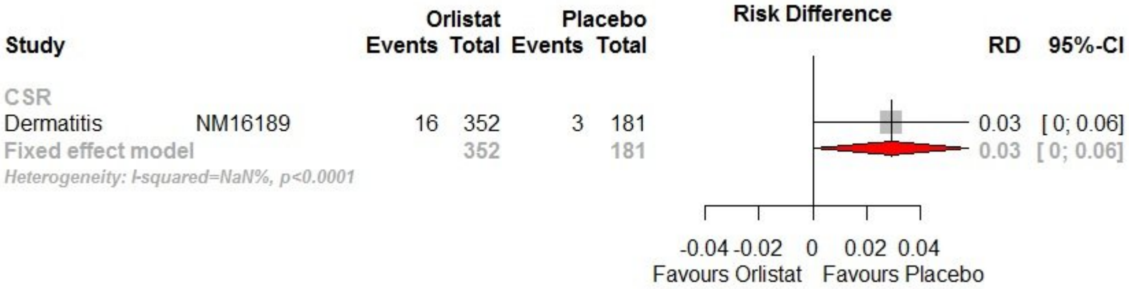

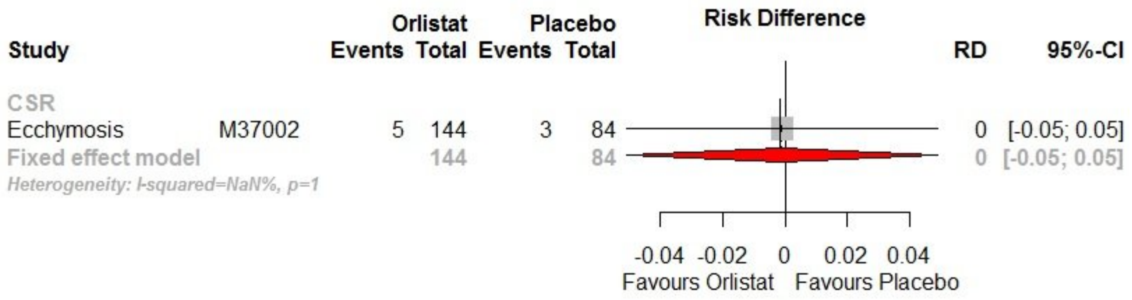

Injury and positioning

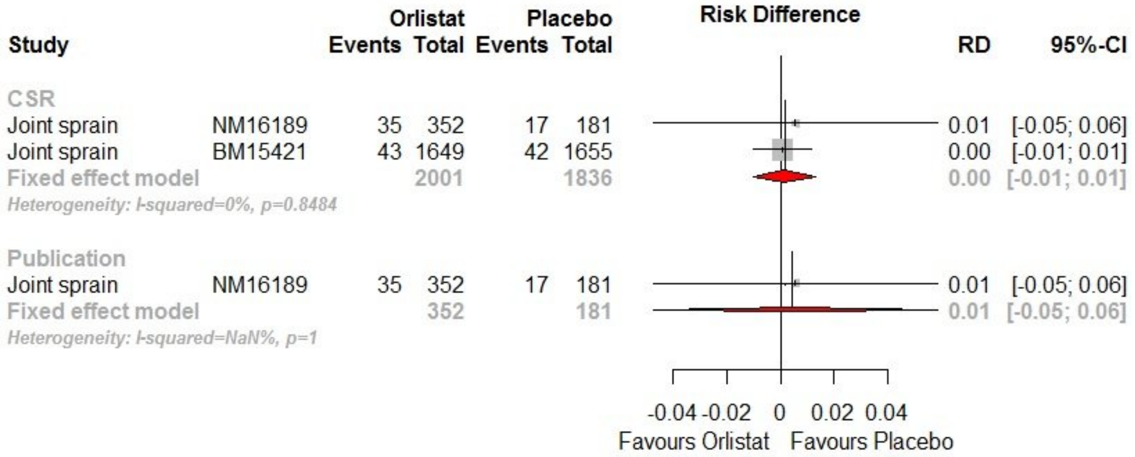

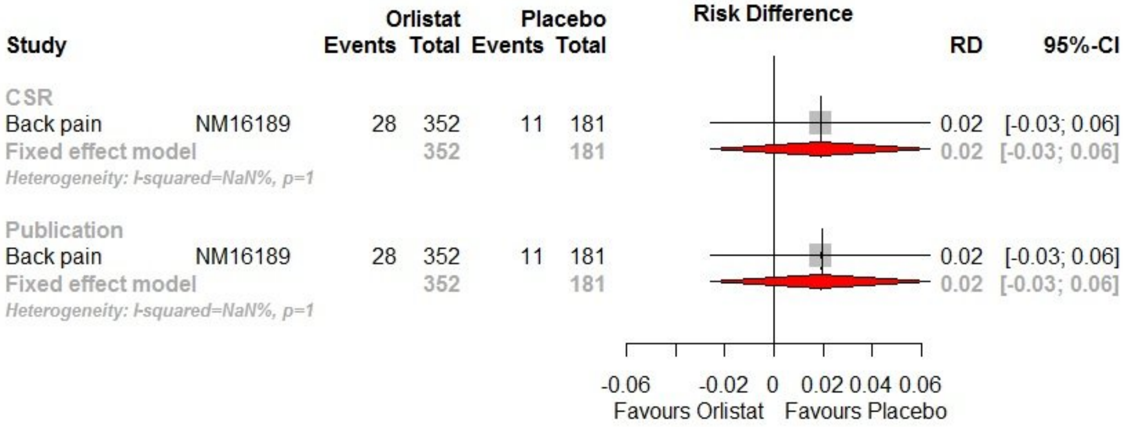

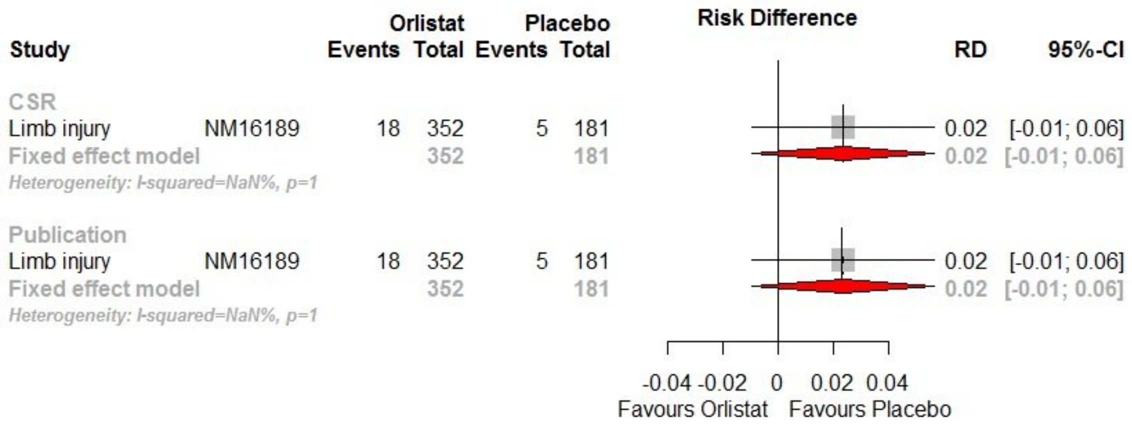

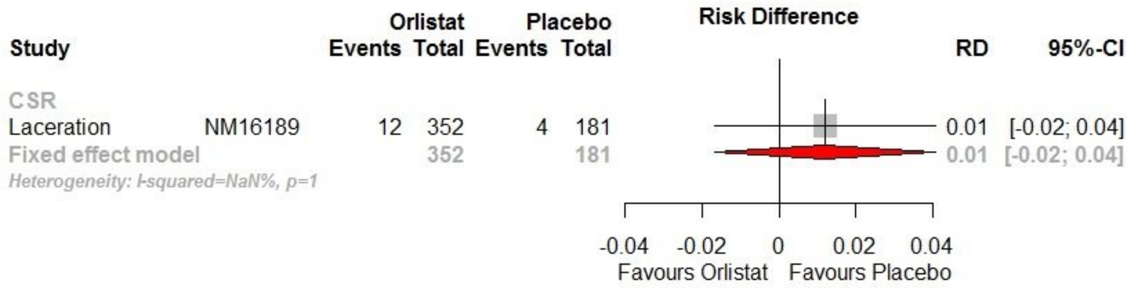

Immune system disorders

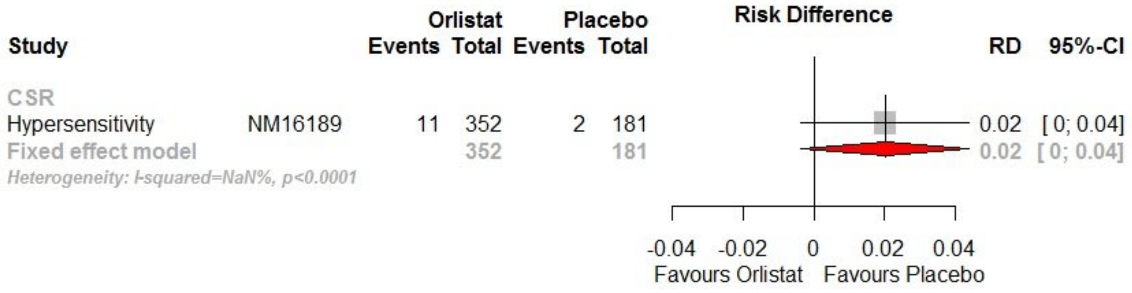

Musculoskeletal, connective tissue and bone disorders

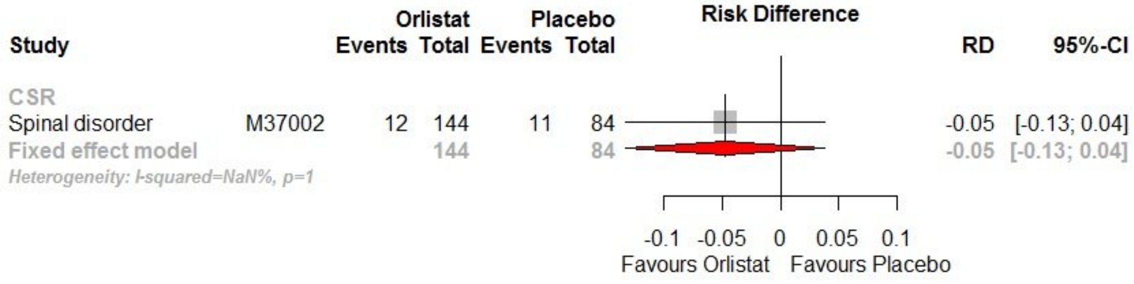

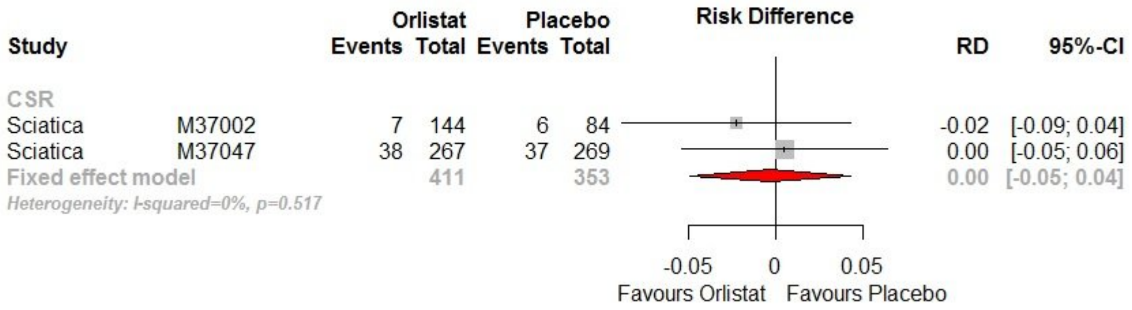

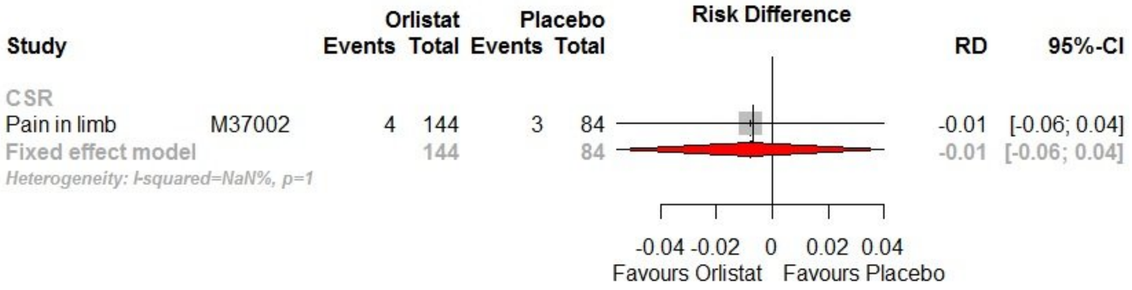

Varicose veins

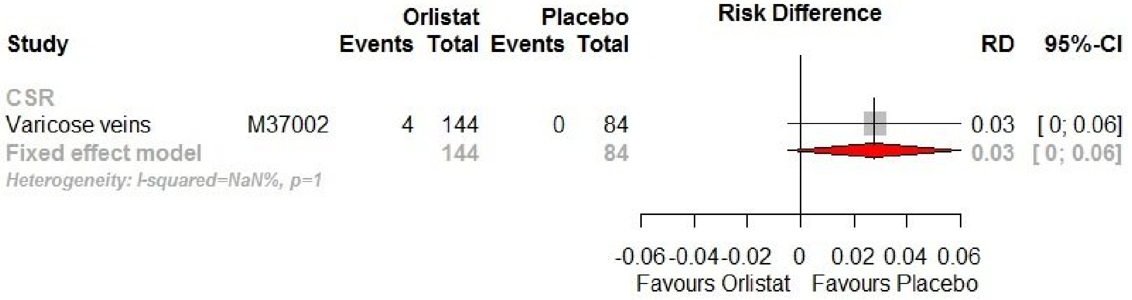

Supplementary Table S1: Search criteria used in both Cochrane central and MEDLINE.

Search set	CENTRAL	MEDLINE
1	(Orlistat or Xenical)	randomized controlled trial.pt.
2		controlled clinical trial.pt.
3		randomized.ab.
4		placebo.ab.
5		clinical trials as topic.sh.
6		randomly.ab.
7		trial.ti.
8		7 or 5 or 2 or 6 or 1 or 4 or 3
9		(orlistat or Xenical).tw.
10		8 and 9
